# SARS-CoV-2 omicron variants harbor spike protein mutations responsible for their attenuated fusogenic phenotype

**DOI:** 10.1038/s42003-023-04923-x

**Published:** 2023-05-24

**Authors:** Seung Bum Park, Mohsin Khan, Sai Chaitanya Chiliveri, Xin Hu, Parker Irvin, Madeleine Leek, Ailis Grieshaber, Zongyi Hu, Eun Sun Jang, Ad Bax, T. Jake Liang

**Affiliations:** 1 Liver Diseases Branch, National Institute of Diabetes and Digestive and Kidney Diseases (NIDDK), National Institutes of Health, Bethesda, MD 20892 USA; 2 Laboratory of Chemical Physics, National Institute of Diabetes and Digestive and Kidney Diseases (NIDDK), National Institutes of Health, Bethesda, MD 20892 USA; 3 National Center for Advancing Translational Sciences (NCATS), National Institutes of Health, Rockville, MD 20850 USA; 4 Department of Internal Medicine, Seoul National University Bundang Hospital, Seoul National University College of Medicine, Seongnam, 13620 Republic of Korea

**Keywords:** SARS-CoV-2, Viral membrane fusion

## Abstract

Since the emergence of the Omicron variants at the end of 2021, they quickly became the dominant variants globally. The Omicron variants may be more easily transmitted compared to the earlier Wuhan and the other variants. In this study, we aimed to elucidate mechanisms of the altered infectivity associated with the Omicron variants. We systemically evaluated mutations located in the S2 sequence of spike and identified mutations that are responsible for altered viral fusion. We demonstrated that mutations near the S1/S2 cleavage site decrease S1/S2 cleavage, resulting in reduced fusogenicity. Mutations in the HR1 and other S2 sequences also affect cell-cell fusion. Based on nuclear magnetic resonance (NMR) studies and in silico modeling, these mutations affect fusogenicity possibly at multiple steps of the viral fusion. Our findings reveal that the Omicron variants have accumulated mutations that contribute to reduced syncytial formation and hence an attenuated pathogenicity.

## Abstract (web summary)

A combination of in vitro, in silico, and biochemical approaches demonstrate that mutations in the S2 domain of the SARS-CoV-2 spike protein are responsible for the attenuated viral fusion phenotype of the Omicron variant BA.1.

## Introduction

The Omicron variant BA.1 of SARS-CoV-2 was first reported in South Africa at the end of 2021 and quickly became a dominant variant of concern (VOC) at the beginning of 2022^1^. Recent data showed that it is correlated with higher case numbers and lower responses to neutralizing antibody^1^. There are five VOCs including Alpha (B.1.1.7), Beta (B.1.351), Gamma (P.1), Delta (B.1.617.2), and Omicron (originally B.1.1.529, reclassified into BA lineages) as well as two variants of interest (VOI) including Lambda (C.37) and Mu (B.1.621) based on the World Health Organization as of December 2021^2^. Among the VOCs, the BA.1 variant and related variants (BA.2, BA.4, BA.5) have emerged as the dominant circulating variants in 2022 and carry more than 30 mutations in its spike protein. For BA.1, over 20 mutations exist in the N-terminal domain and receptor binding domain (RBD). The next 5 mutations (T547K, D614G, H655Y, N679K, P681H) are located in the subdomain 1/2 or near the S1/S2 cleavage site (R685) and the last 6 mutations (N764K, D796Y, N856K, Q954H, N969K, and L981F) are positioned around the S2’ cleavage site (R815) and HR1 (heptad repeat 1) region^3^.

It has been reported that the spike proteins of the B.1.1.7 and B.1.617.2 variants are more efficient in cell-cell fusion compared to the Wuhan strain due to mutations located near the furin cleavage site that improve S1/S2 cleavage efficiency^4,5^. The higher pathogenicity of the B.1.1.7 and B.1.617.2 variants are associated with more efficient syncytial formation because virus-induced syncytial formation has been linked with pathogenesis and disease severity^6^. On the other hand, BA.1 seems to be clinically more infectious compared to the other VOCs and recent data revealed that the BA.1 variant has a higher reinfection and vaccine breakthrough rate, probably because of its resistance to pre-existing neutralizing antibodies^1,7^. Interestingly, the BA.1 variant is associated with less severe disease compared to the B.1.617.2 variant^8^. Based on the relationship between syncytial formation and pathogenesis, it was hypothesized that the BA.1 variant may have a lower syncytial formation capacity. However, subsequent studies regarding syncytial formation have been controversial^9,10^. Since mutations near the furin cleavage site of B.1.1.7 and B.1.617.2 variants improved S1/S2 cleavage efficiency, we hypothesize that the reduced syncytial formation of BA.1 variant may be a result of mutations near the furin cleavage site resulting in less efficient S1/S2 cleavage.

Recent studies have reported on mutations affecting epitopes of neutralizing antibodies and spike protein’s RBD in BA.1 variants^11,12^. Here we examined the biological properties of BA.1 variants and focused mainly on spike-mediated fusion using cell-cell fusion assays. We showed that attenuated syncytial formation is not only due to mutations near the furin cleavage site, but also mutations in and around the HR1 region. While many of the mutations affect the S1/S2 cleavage, some mutations in the HR1 region seem to directly affect the virus-induced fusion process. Together these mutations plausibly explain the altered pathogenic phenotype of the BA.1 variants.

## Results

### Fusogenic activities of SARS-CoV-2 variants and responses to inhibitors and neutralizing antibody

**Fig. 1:**
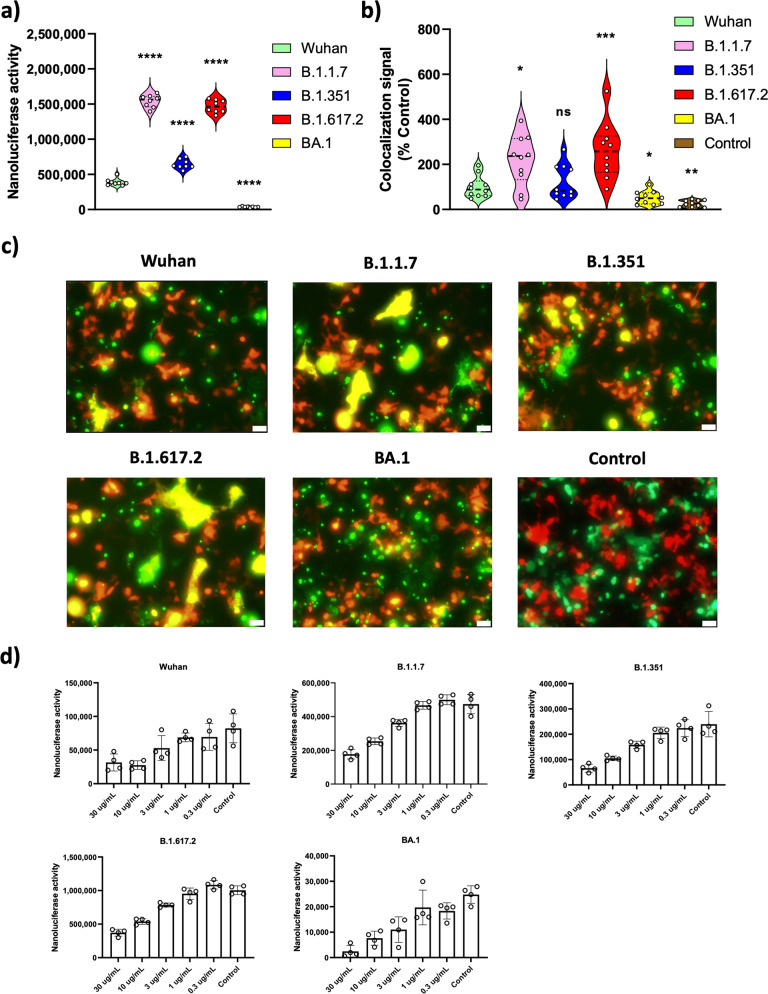
Fusogenic activities of SARS-CoV-2 variants and responses to neutralizing antibody. **a** Cell-cell fusion assay was performed with SARS-CoV-2 variants S (see Methods). The various truncated S-SmBit transfected donor (HeLa) cells and the LgBit transfected recipient (293ACE2) cells were mixed 24 h post-transfection and were incubated for 48 h. After incubation, luminescence signals were measured by a POLARstar Omega plate reader. All data points are presented as mean values (standard deviation (SD), *n* = 8 biological independent replicates). **b**, **c** Similarly, the various S-GFP transfected donor (HeLa) cells and the RFP transfected recipient (293ACE2) cells were mixed 24 h post-transfection and incubated for an additional 48 h. After incubation, colocalization signals between GFP and RFP were measured using CellSens fluorescence microscope. GFP-SmBit expressing plasmid was transfected into donor (HeLa) cells for control. For quantification, 10 fields were randomly selected to measure the fusion activity (*n* = 10). Colocalization signals were quantified by ImageJ. For statistical comparison, adjusted *P* values are shown (vs Wuhan, *P < 0.05; **P < 0.01; ***P < 0.001; ****P < 0.0001; ns: not significant). All results are representative of three independent experiments. Scale bar 50 µm. **d** The various truncated S-SmBit transfected donor (HeLa) cells and the LgBit transfected recipient (293ACE2) cells were mixed 24 h post-transfection and were treated with five different concentrations (30 µg/mL, 10 µg/mL, 3 µg/mL, 1 µg/mL, 0.3 µg/mL) of anti-S antibody. 48 h postmixture, luminescence signals were measured using a POLARstar Omega plate reader. All data points are presented as mean values (SD, *n* = 4 biological independent replicates).

The aligned spike protein sequences of all major SARS-CoV-2 variants show that many mutations are far more commonly present throughout the spike sequences of the BA.1 and related variants compared to other variants, including many affecting the S2 domain, which mediates cell-cell fusion after receptor binding by the S1 domain (Supplementary Fig. 1**)**. Previous studies suggested that the BA.1 variant has a lower fusogenicity than the ancestral strain (Wuhan) of SARS-CoV-2^10,13^. To confirm the fusogenicity of BA.1 and earlier lineages, including Wuhan, B.1.1.7, B.1.351, and B.1.617.2, we performed cell-cell fusion assays using the SmBit-LgBit (split luciferase) and the GFP-RFP systems that were previously developed in our laboratory^14^. We also tried to constitutively express S protein using recombinant lentiviruses and selecting S-expressing construct transfected cells by antibiotic marker earlier, but those strategies were unsuccessful. However, since our previously developed transient transfection system ensured high transfection efficiency, we decided to use this system for the rest of the experiments. Both C-terminal truncated (more efficient cell surface localization) and full-length S constructs were tested in these systems. Briefly, donor cells (HeLa cells) express S-SmBit or S-GFP fusion protein and recipient cells (293ACE2) express LgBit or RFP. Since HeLa cells do not express ACE2^15^, they do not undergo self-fusion. Confocal microscopy demonstrates similar expression of spike proteins in the donor HeLa cells (Supplementary Fig. 2a). After successful fusion between donor and recipient cells, luminescent signals can be detected due to SmBit and LgBit interaction and yellow fluorescence signals from GFP and RFP colocalization. As expected, we observed BA.1 showing significantly lower fusogenicity in both the SmBit-LgBit (Fig. 1a) and the GFP-RFP systems (Fig. 1b, c). The GFP-RFP colocalization signals were quantified by ImageJ and shown in Fig. 1b. Overall, the C-terminal truncated S variants showed higher fusogenicity compared to untruncated S variants as expected (Supplementary Fig. 3).

A neutralizing polyclonal anti-S antibody which targets the S2 domain blocked the fusion of all SARS-CoV-2 variants similarly (Fig. 1d). Previously described fusion inhibitors such as 25-hydroxycholesterol (25-HC), itraconazole, and dichlorcyclizine showed similar dose-dependent inhibition of fusion activity of SARS-CoV-2 variants (Supplementary Fig. 4)^14,16^. We also confirmed that the different fusogenic activities of SARS-CoV-2 variants are supported by the comparable total spike protein expression levels between the variants by Western blot assay (Fig. 4a) and the similar cellular distribution patterns between the variants observed by confocal microscopy (Supplementary Fig. 2a). We next tested potential cytotoxicity due to spike protein expression and did not observe any significant differences among the variants (Supplementary Fig. 2b). Additionally, we measured infectivity of variants using SARS-CoV-2 S coated vesicular stomatitis virus (VSV) pseudoparticles and found that B.1.1.7, B.1.351, B.1.617.2, and BA.1showed overall higher infectivity than the ancestral Wuhan strain (Supplementary Fig. 5). Infectivity of infectious BA.1 appeared to be dependent on cell lines tested^10,17,18^. Interestingly, even though the fusogenicity of BA.1 was substantially lower than Wuhan, its infectivity was comparable if not higher than Wuhan in our system. The higher infectivity of BA.1 is likely due to mutations in the S1 region, such as the receptor binding domain, that confer higher affinity to ACE2^10^. The combined effect of higher receptor engagement and lower fusogenicity may still result in a higher infectivity.

### Live virus fusion assay of SARS-CoV-2 variants

**Fig. 2:**
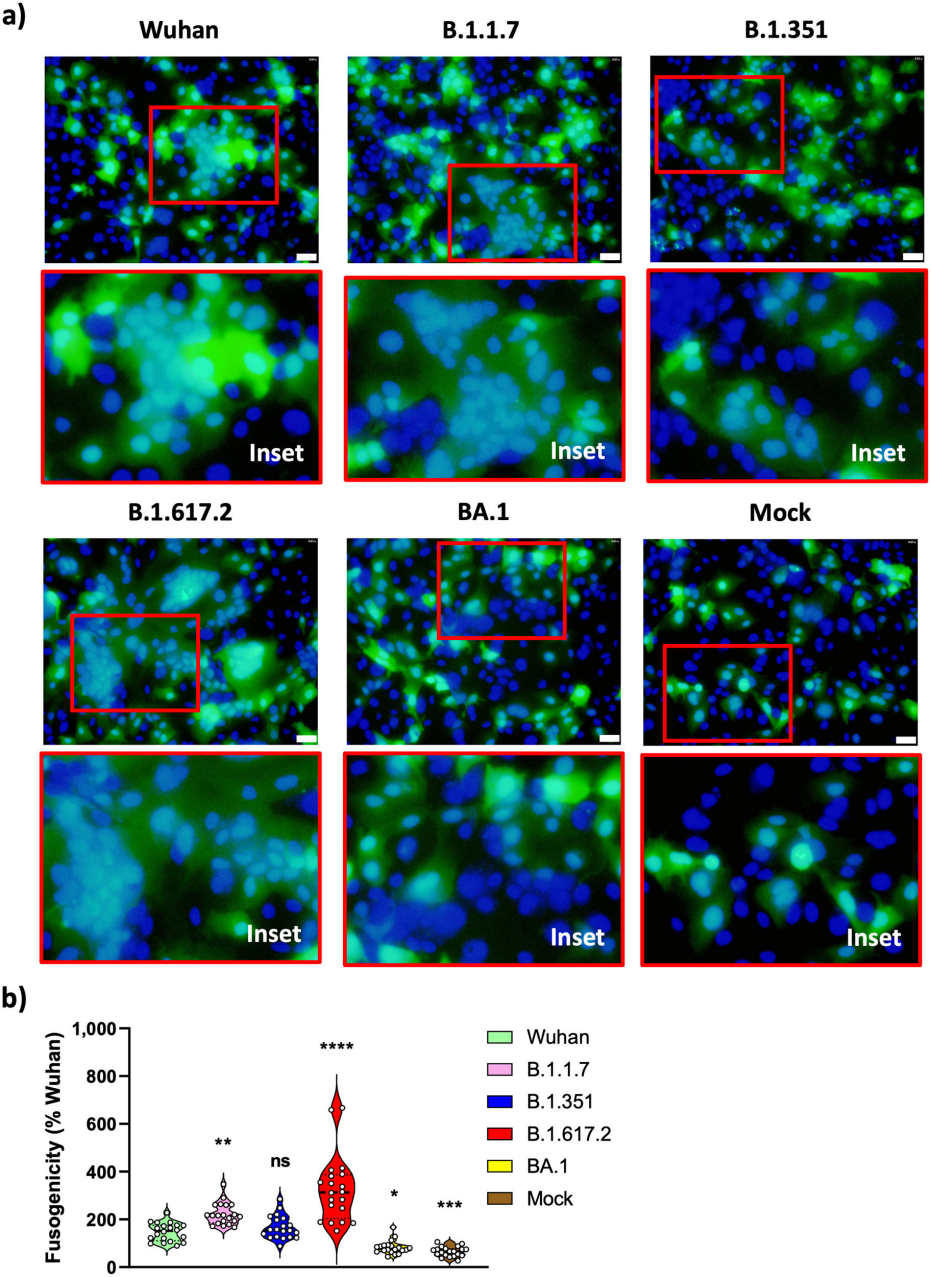
Live virus fusion assay of SARS-CoV-2 variants. **a** Huh7.5-A2T2 cells expressing GFP were seeded in 96-well plates. 24 h postseeding, the cells were infected with SARS-CoV-2 variants and incubated for 2 h. The cells were washed after 2 h and incubated for another 22 h followed by 4% paraformaldehyde (PFA) fixation. After incubation, DAPI was used for nuclei staining. GFP and DAPI signals were evaluated using CellSens fluorescence microscope. **b** For quantification, 20 fields were randomly selected to measure the fusion activity (*n* = 20). Fusogenicity was quantified by the number of DAPI nuclei divided by the number of GFP blobs. For counting the number of GFP blobs, one continuous GFP blob was counted one fused or unfused cell. When there is discontinuous GFP signal intensity or discontinuous circular blob-shaped GFP signal in the blob, then they were counted separately to distinguish unfused neighboring cells from fused cells. The average numbers of DAPI nuclei and GFP blobs per location are followed; Wuhan (DAPI: 103.7, GFP: 19.4), B.1.1.7 (DAPI: 98.3, GFP: 12.0), B.1.351 (DAPI: 86.4, GFP: 14.3), B.1.617.2 (DAPI: 122.6, GFP: 11.0), BA.1 (DAPI: 68.6, GFP: 23.0), Mock (DAPI: 68.2, GFP: 29.0). For statistical comparison, adjusted *P* values are shown (vs Wuhan, *P < 0.05; **P < 0.01; ***P < 0.001; ****P < 0.0001; ns: not significant). All results are representative of three independent experiments. Scale bar 50 µm.

A previous study showed that SARS-CoV-2-infected cells express the spike protein at their cell surface and form syncytia with neighboring ACE2-positive cells^19^. To confirm syncytial formation in SARS-CoV-2-infected cells and to test the fusogenicity of various SARS-CoV-2 variants, we performed a live virus fusion assay. For this assay, a Huh7.5 cell line stably expressing ACE2 and TMPRSS2 (Huh7.5-A2T2) was transfected with GFP plasmid and selected by antibiotic G418. Then, this stable cell line was infected with various SARS-CoV-2 variants. 24 hours after infection, syncytial formation was detected (Fig. 2a). The packed 4’,6-Diamidino-2-Phenylindole dilactate (DAPI, nuclei) staining is represented as a syncytium (GFP blob) and is enlarged in each inset picture. Higher fusogenicity was detected in B.1.1.7 and B.1.617.2 variants-infected cells and lower fusogenicity was shown for the BA.1 variant. B.1.351 variant did not demonstrate significant difference compared to the Wuhan strain. For quantification, 20 fields were randomly selected and the fusogenicity was quantified by the number of DAPI-stained nuclei divided by the number of GFP blobs (Fig. 2b). Taken together, SARS-CoV-2 variants showed similar fusogenic behavior as the findings from the S-triggered cell-cell fusion assays reported in Fig. 1a, b.

### Identification of mutation(s) for the reduced fusogenicity of BA.1

**Fig. 3:**
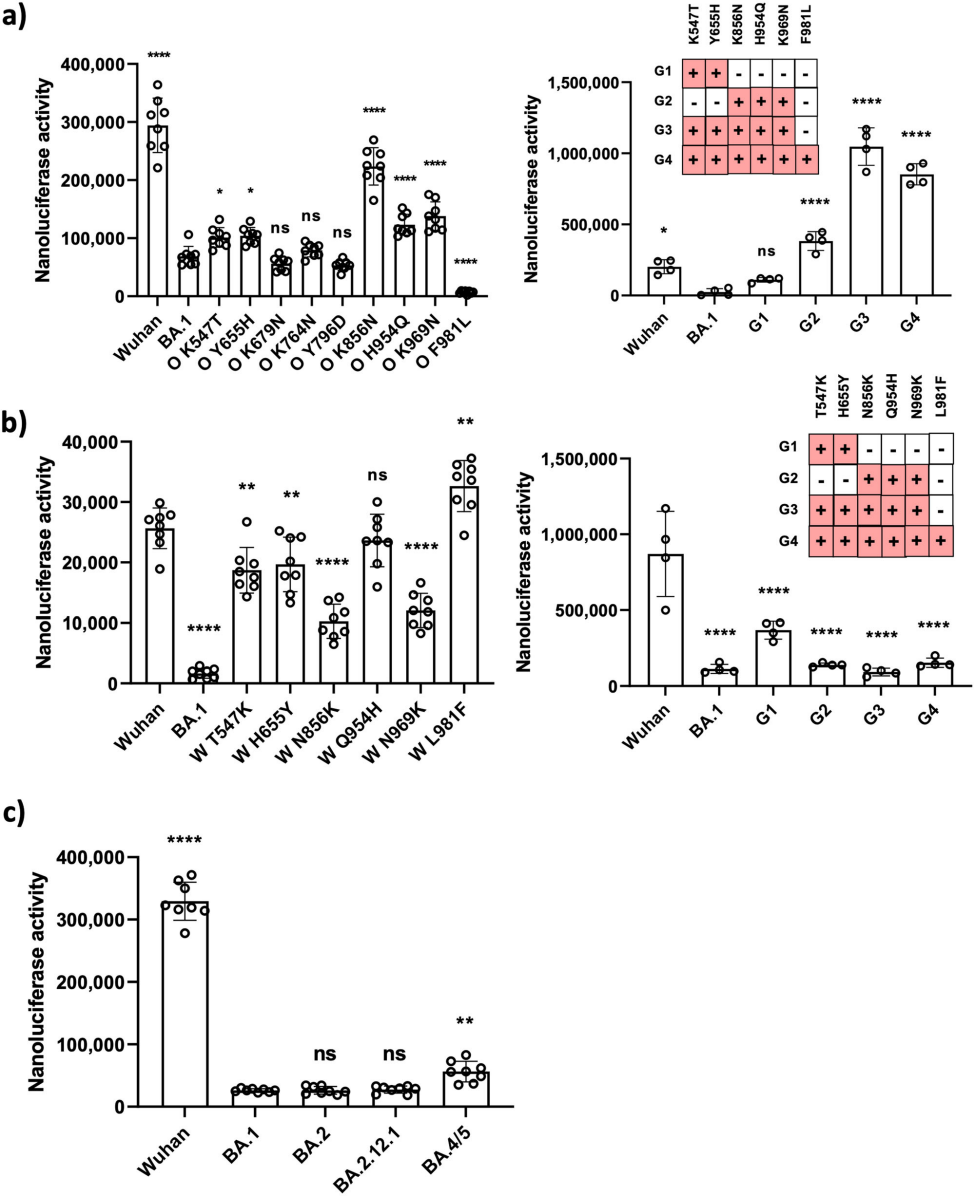
Identification of mutation(s) for the reduced fusogenicity of BA.1. Cell-cell fusion assay was performed with BA.1-associated S mutants. The various truncated S-SmBit transfected donor (HeLa) cells and the LgBit transfected recipient (293ACE2) cells were mixed 24 h post-transfection and were incubated for 48 h. After incubation, luminescence signals were measured by a POLARstar Omega plate reader. **a** Individual mutations were introduced into the BA.1 S sequence. Except for the corresponding mutations, the rest of the sequence is same as the native BA.1 S sequence. Similarly, the combination mutations were also introduced into the BA.1 S sequence. All data points are presented as mean values (SD, *n* = 4-8 biological independent replicates). For statistical comparison, adjusted *P* values are shown (vs BA.1, *P < 0.05; ****P < 0.0001; ns: not significant). All results are representative of three independent experiments. **b** Individual mutations were introduced into the Wuhan S sequence. Except for the corresponding mutations, the rest of the sequence is same as the native Wuhan S sequence. Similarly, the combination mutations were also introduced into the Wuhan S sequence. All data points are presented as mean values (SD, *n* = 4-8 biological independent replicates). For statistical comparison, adjusted *P* values are shown (vs Wuhan, **P < 0.01; ****P < 0.0001; ns: not significant). All results are representative of three independent experiments. **c** The fusogenic activities of various BA.1 subvariants S constructs were measured similarly. All data points are presented as mean values (SD, *n* = 8 biological independent replicates). For statistical comparison, adjusted *P* values are shown (vs BA.1, **P < 0.01; ****P < 0.0001; ns: not significant). All results are representative of three independent experiments.

To investigate the effects of mutations in the BA.1 variant, we introduced individual or combination mutation(s) in Wuhan or BA.1 spike sequences. First, we introduced individual mutations in the BA.1 spike sequence. Among more than 30 mutations in the BA.1 variant (Supplementary Fig. 1), we decided to pursue the C-terminal 11 mutations, which likely affect fusion activity because of their proximity to S1/S2, S2’ cleavage sites, and HR1 region. Two mutations among these 11 mutations (D614G and P681H) have already been characterized in previous studies^20–22^. We thus investigated the other 9 mutations. These 9 mutations were introduced into the BA.1 spike sequence individually to revert each mutation back to the Wuhan sequence. Among them, reverse mutations at amino acid 547 (K547T), 655 (Y655H), 856 (K856N), 954 (H954Q), and 969 (K969N) showed higher fusogenicity compared to the original BA.1 variant, suggesting that these mutations are responsible for the decreased fusogenicity of BA.1 variant (Fig. 3a). Interestingly, the reverse mutation at amino acid 981 (F981L) exhibited lower fusogenicity compared to the BA.1 variant, suggesting that this mutation may have emerged to compensate for other decrease-of-function mutations in the BA.1 variant (Fig. 3a). Three other mutations K679N, K764N, and Y796D did not show any appreciable differences in fusion activity from the BA.1 variant.

Next, we tested combined mutations with one set near the S1/S2 cleavage site, and the other near the S2’ cleavage site and in HR1 region. K547T and Y655H were introduced together into one construct, and K856N, H954Q, and K969N were combined separately in another construct. As expected, combination mutants showed higher fusogenicity compared to the individual mutants. When the 5 mutations were combined altogether, the fusogenicity was even higher than the Wuhan spike construct. As expected, the fusogenicity was reduced when the putative compensatory F981L was introduced into the 5 combined mutations (Fig. 3a). Interestingly, the mutant containing K856N, H954Q, and K969N showed higher fusogenicity than the one containing K547T and Y655H, suggesting that the mutations near the S2’ cleavage site and in HR1 region have a stronger effect on fusogenicity than the mutations near the S1/S2 cleavage site.

To verify the effects of the above 6 mutations, we introduced individual or combination BA.1 mutations into the Wuhan spike sequence. Since they are reverse mutations of above mutations, we expected them to have an opposite fusogenicity pattern. Indeed, mutations at amino acid 547 (T547K), 655 (H655Y), 856 (N856K), and 969 (N969K) showed lower fusogenic activities compared to the Wuhan strain, supporting that these mutations are responsible for the decreased fusogenic phenotype of the BA.1 variant. As expected, mutation at amino acid 981 (L981F) exhibited higher fusogenicity than the Wuhan strain. Mutation at amino acid 954 (Q954H) did not show any significantly reduced fusogenicity compared to the Wuhan strain (Fig. 3b). It is possible that this mutation may require the presence of other mutations to exhibit its effect when it is introduced into the Wuhan strain. Again, the combination mutations demonstrated significantly lower fusogenicity than the Wuhan strain in a pattern that is consistent with the previous mutational analyses in the BA.1 variant.

To examine further the fusogenicity of other BA.1 subvariants, including BA.2, BA.2.12.1 and BA.4/5, we generated the BA.1 subvariant spike sequences shown in Supplementary Fig. 1. Because BA.4 and BA.5 share the same spike sequence, we label it as BA.4/5. In the cell-cell fusion assay, BA.4/5 showed slightly increased fusogenicity and BA.2 and BA.2.12.1 showed no significant difference from the BA.1 variant (Fig. 3c).

Taken together, we identified 5 individual mutations that are responsible for the decreased fusogenic phenotype of the BA.1 variant, and one compensatory mutation that is associated with increased fusogenicity. Also, mutations in or around the S2’ cleavage site and HR1 may have a stronger effect on fusogenicity compared to mutations near the S1/S2 cleavage site. Lastly, all BA.1 subvariants except for BA. 4/5 have comparable fusogenicity.

### Effects of BA.1 variant mutations on S1/S2 cleavage

**Fig. 4:**
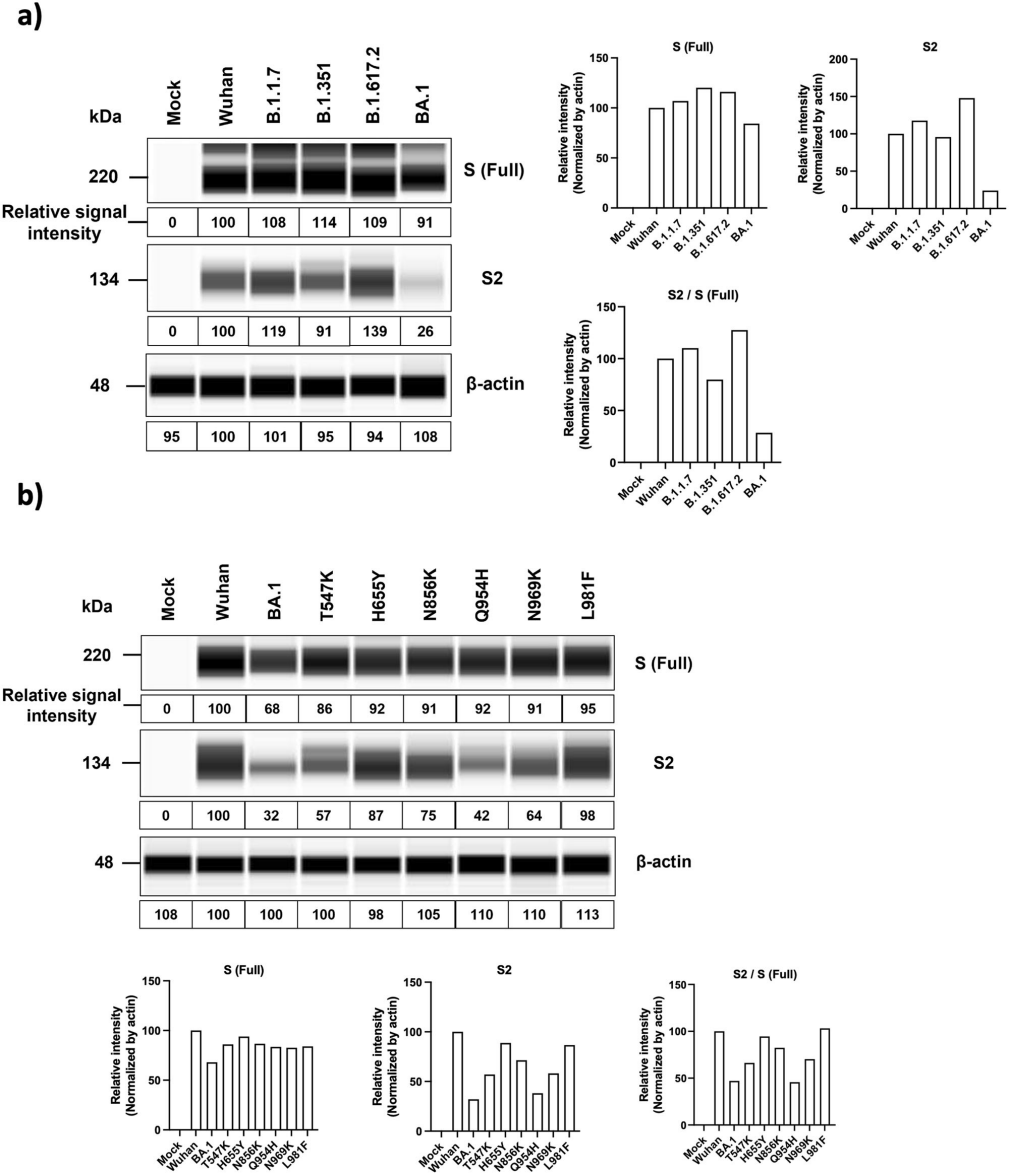
Effects of BA.1 variant mutations on S1/S2 cleavage. Huh7.5-A2T2 cells were transfected with SARS-CoV-2 variants S expressing vectors (**a**) or BA.1-associated mutants S expressing vectors (**b**). 24 h post-transfection, cells were collected with RIPA Lysis and Extraction Buffer and Protease Inhibitor Cocktail. The lysed samples were processed further to measure the S/S2 level with automated Western blot system (Simple Western™ Automated Western Blot System). Then, ImageJ was used to quantify the relative signal intensity. The β-actin-normalized relative signal intensities were plotted separately as S (Full), S2, and S2/S (Full) ratio. The data are representative of three independent experiments.

Increased cleavage of S1/S2 site has been associated with increased infectivity, fusogenicity and pathogenicity of SARS-CoV-2 variants^23,24^. Here we aim to determine whether the BA.1 mutations identified above may result in reduced S1/S2 cleavage accounting for the decreased fusogenicity. We tested the S1/S2 cleavage of SARS-CoV-2 variants and various BA.1-associated spike mutants. We transfected these spike-expressing plasmids into Huh7.5-A2T2 and analyzed levels of full-length spike and S2 (Fig. 4). The spike protein levels were comparable among the Wuhan, B.1.1.7, B.1.351, and B.1.617.2 variants. The BA.1 spike protein level appeared slightly lower than the other variants (Fig. 4a). B.1.1.7 and B.1.617.2 showed higher S2 levels, while BA.1 exhibited lower S2 level than Wuhan (Fig. 4a). This difference in S1/S2 cleavage is similar to the pattern observed in cell-cell fusion assay in most of the variants with the exception of the Q954H mutation, BA.2, BA.2.12.1, and BA.4/5 which are all BA.1 subvariants showed similar S2 levels like the one of BA.1 and much lower than the one of Wuhan (Supplementary Fig. 6a). Testing of individual BA.1 mutations showed variably reduced S1/S2 cleavage, which is reproducible in two other cell lines (Fig. 4b and Supplementary Fig. 6b-c).

### Effect of BA.1 mutation Q954H on lipid-bound state of spike protein during fusion

**Fig. 5:**
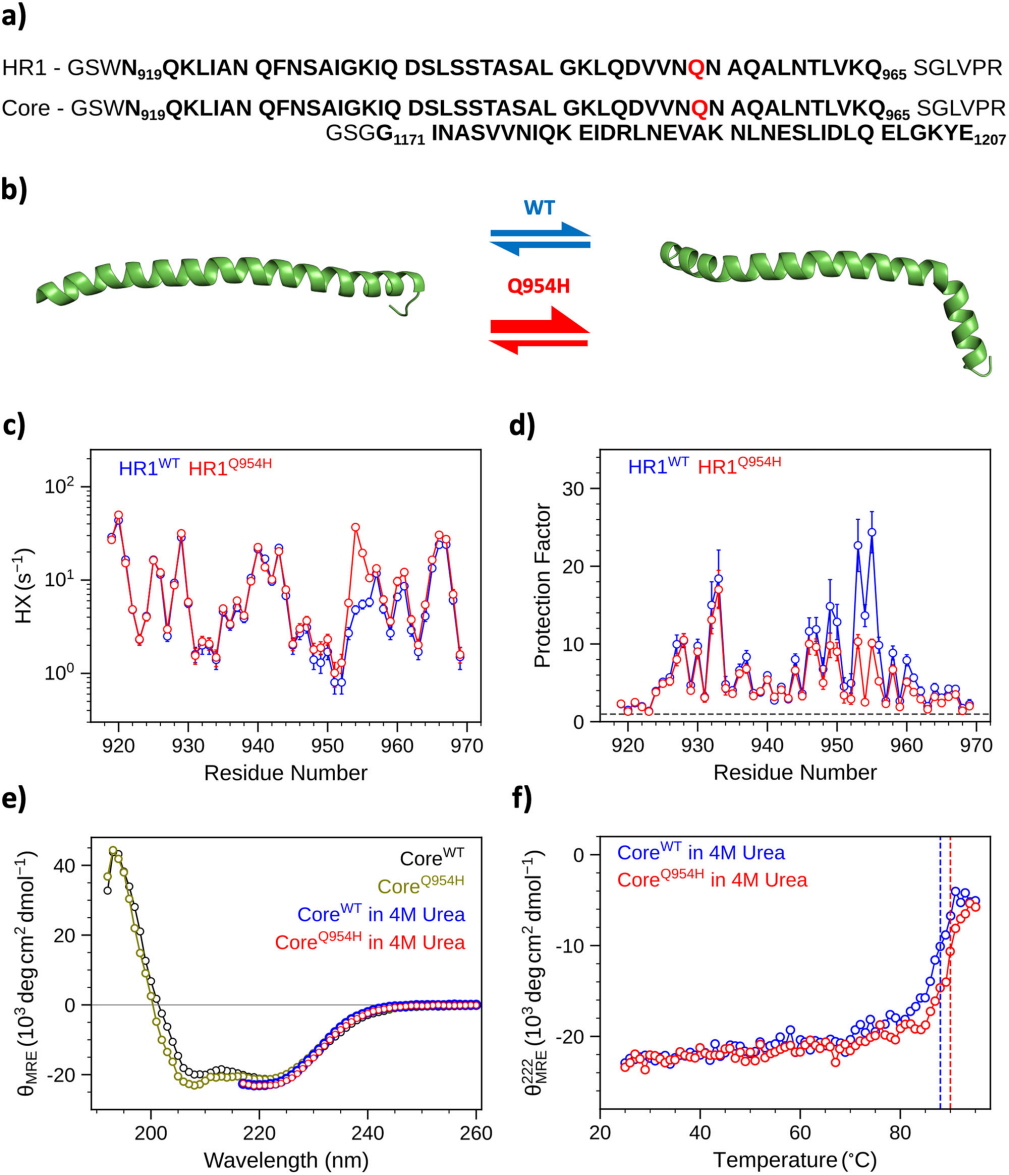
Effect of BA.1 mutation Q954H on lipid-bound state of spike protein during fusion. **a** The primary amino acid sequence of HR1 (residues N919-Q965) and HR2 (residues G1171-E1207) in bold. In the Core construct a short flexible linker sequence (SGLVPRGSG) connects the HR1 and HR2 helical repeats. The site of mutation, Glu-954 is shown in red. **b** Ribbon representations for the two (straight and kinked) lipid-bound conformations of HR1 that are in equilibrium and fast exchange with each other. The Q954H mutation increases the population of the kinked conformation. **c** Residue-specific HX rates and (**d**) the corresponding protection factors of HR1^WT^ (blue) and HR1^Q954H^ (red), normalized at pH 7. A protection factor of 1 (dashed line) corresponds to no protection from hydrogen exchange, i.e., no significant population of intramolecular hydrogen bonds. Data were obtained on 100 µM [^15^N/^2^H]-HR1^Q954H^ in the presence of 100 mM dimyristoyl phosphatidylcholine (DMPC)/dihexanoyl phosphatidylcholine (DHPC) (q ~ 0.5). Data for HR1^WT^ is reproduced from earlier work^25^. **e** Overlay of Far-UV CD spectra of 10 µM Core in the absence (Core^WT^-black and Core^Q954H^-olive) in the presence of 4 M Urea (Core^WT^-blue and Core^Q954H^-red). **f** Thermal melting of Core^WT^ (blue) and Core^Q954H^ (red) as observed by CD in the presence of 4 M Urea. Melting temperatures of Core^WT^ and Core^Q954H^ are 88 °C and 90 °C, respectively.

Since the three mutations, Q954H, N969K, and L981F, reside in the HR1 region, they may affect the viral fusion process. We therefore carried out a more detailed structural study of one of these HR1 mutations. Previously, we have shown that the HR1 region (residues N919-Q965) adopts a membrane-bound helical conformation in the presence of bilayer mimetics (bicelles) that likely represents a lipid-bound intermediate state of the viral membrane fusion process^25^. In this lipid-bound state, the α-helical structure adopted by the amphipathic HR1 samples at least two conformations that are in rapid exchange with one another: one set of the conformers adopts a regular straight a-helix, and the other contains a kink at the Q954 amino acid position (Fig. 5b). Because Q954H corresponds to one of the BA.1 variants, we searched for any structural changes associated with this mutation, both in the lipid-bound state and the post-fusion six-helical bundle (6HB) state. We previously measured the backbone amide hydrogen exchange (HX) rates for the wild-type (WT) sequence in the lipid-bound state at high precision. Backbone amide protons exchange with solvent when not engaged in stable intra- or intermolecular hydrogen bonds^26^, i.e., this exchange rate indicates the fraction of time that H-bonds are transiently disrupted. For most of HR1, HX rates for the Q954H mutant are very similar to those of the corresponding WT HR1 residues, suggesting similar dynamic properties of the mutant and the WT (Fig. 5c). Because HX rates are sensitive to the type of residue and its immediately preceding neighbor^27^, HX rates for the WT and the mutant can differ at the site of mutation. To remove this residue-type dependence, the HX rates were converted to protection factors, which removes the dependence on residue type. Again, for most of the polypeptide chain of HR1 protection factors were found to be very similar for the WT and Q954H mutant (Fig. 5d). However, about a three-fold decrease in the protection factors is observed for the Q954H mutant at the N953-N955 region, suggesting that the mutant favors the formation of the kinked conformation. A much smaller*, ca* 15% decrease in HX protection extends from residue A944 to K964. Because both straight and kinked helical conformations are lipid-bound, the HX measurement, together with only very small differences in chemical shifts relative to WT (Supplementary Fig. 7), indicates that the Q954H mutation does not significantly alter the lipid-binding propensity of HR1. The increase in chemical shift changes for the C-terminal region (L959-V969) appears to be linked to the shift in the population towards the kinked conformer for the Q954H variant.

Consistent with a previously reported cryo-EM study^28^, we found that in solution the structure of the 6HB, post-fusion state, formed by the Core construct (HR1 and HR2 connected by a short flexible linker) is not significantly impacted by the Q954H mutation^29^. The far-UV CD spectra of both the WT and Q954H of the 6HB state display the same characteristic α-helical signature with two minima at 208 nm and 222 nm (Fig. 5e). To rule out any difference in thermodynamic stability between the WT and Q954H mutant, thermal melting curves were monitored at 222 nm. As the actual melting temperatures were far above 100 °C, experiments were conducted in the presence of a denaturant to obtain a quantitative comparison of the two constructs. Interestingly, even in the presence of rather high concentrations of denaturant, 4 M urea, the secondary structure of the 6HB appears largely unperturbed for both the WT and the mutant (Fig. 5e). Melting temperatures of 88 and 90 °C were measured for the WT and Q954H, suggesting no significant difference in stability of the post-fusion 6HB states between the two constructs (Fig. 5f).

A recent paper reported the requirement of acidic pH for SARS-CoV-2 to successfully infect cells^30^. Therefore, we investigated whether H954, which can change its protonation state during acidification, is engaged in pH-dependent 6HB-stabilizing interactions that involve its imidazole moiety (Supplementary Fig. 8). Such an interaction would result in deviation of its pK_a_ value from 6.5, which applies in the absence of structure-stabilizing interactions^31^. The experimentally determined value for H954, pK_a_ = 6.4, indicates the absence of such an interaction for the 6HB state across the pH range relevant for membrane fusion.

### Effects of mutations on viral fusion by in silico modeling

**Fig. 6:**
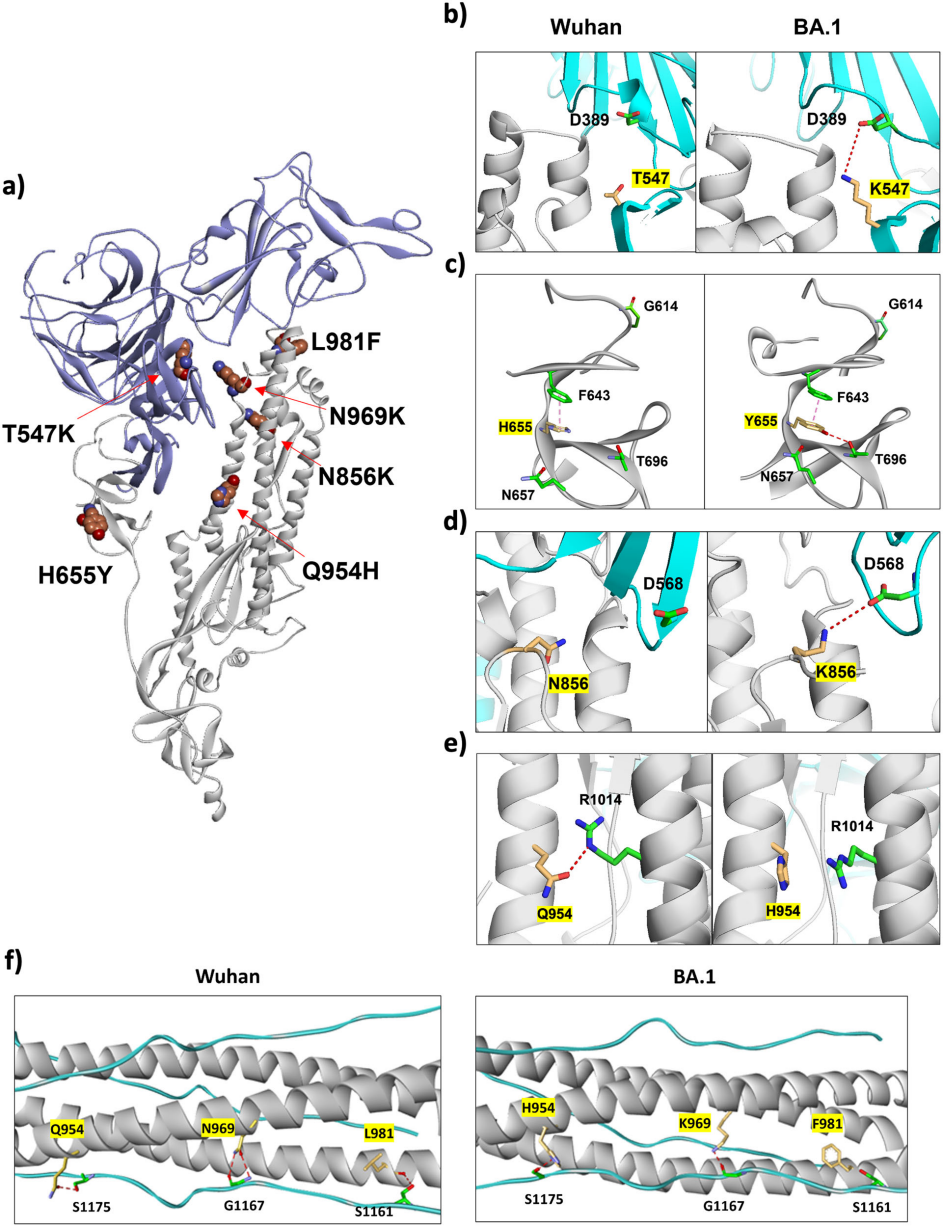
Effects of mutations on viral fusion by in silico modeling. **a** Ribbon representation of the BA.1 spike monomer protein in the close conformation. S1 domain is shown in blue and S2 domain is shown in grey. The mutations are rendered in balls and colored in red, blue and brown for the O, N, and C atoms, respectively. **b** Mutation of T547K in the WT and BA.1. The trimer proteins are shown in ribbons in grey (S2) and cyan (S1). The H-bond formed between K547 and D389 in BA.1 is shown in red dotted line. **c** Mutation of H655Y at the S1/S2 cleavage site (G614). The aromatic stacking interaction between H655Y and F643 is shown, whereas an additional H-bond is formed between Y655 and T696 in the BA.1. **d** Mutation of N856K observed in the Wuhan and BA.1 variant. K856 forms a salt bridge with D568. **e** Mutation of Q954H in the Wuhan and BA.1. Q954 but not H954 forms an H-bond with R1014. **f** Mutations of Q954H, N969K, and L981F in the HR1 domain (grey) and form H-bond interactions with residues of the HR2 domain (cyan) in the postfusion 6HB structure.

The structures of SARS-CoV-2 spike protein in both prefusion and postfusion states have been resolved by cryo-EM^32^. In addition, the 6HB structure of HR1 and HR2 in the postfusion state has been further characterized for the variants^28^. Based on these structures, we modeled the 6 mutations described above and evaluated their potential effects on the structure and function of the spike protein in the context of viral fusion (Fig. 6). The locations of these mutations are shown on the monomeric structure of S (Fig. 6a). The T547K mutation locates in the subdomain 1/2 and forms a salt bridge with D389 (Fig. 6b). This effect may alter the S1/S2 interaction and affect S1/S2 cleavage, like the D614G and P681H mutations^20,22,24^. The H655Y mutation near the S1/S2 cleavage site can form multiple interactions with other adjacent amino acids (Fig. 6c), which likely confers altered S1/S2 cleavage. It is also possible that this mutation may affect the subsequent S2’ cleavage after receptor engagement. The N856K mutation in the S2 domain can form a salt bridge with D568 of S1, possibly affecting S1/S2 interaction and reducing S1/S2 cleavage (Fig. 6d). The Q954H mutation locates in the HR1. The wildtype glutamine but not the histidine mutation can form a hydrogen bond with R1014 (Fig. 6e), which may cause a subtle conformational change of the S2 domain and result in a less efficient S1/S2 cleavage. The N969K mutation in the HR1 points to solvent and does not make any interactions with other amino acids in the prefusion trimer structure. However, this mutation can still decrease S1/S2 cleavage (Fig. 4). It is possible that this mutation may have a subtle effect on the conformation of S2 with resulting lower S1/S2 cleavage efficiency. The L981F mutation in the distal region of HR1 has no discernable effect on intermolecular interaction. Thus, we don’t have a good explanation for its effect on S1/S2 cleavage.

Finally, the Q954H, N969K, and L981F mutations reside in the HR1 domain that together with the HR2 forms the 6HB in the post-fusion structure. It is possible that they may affect the 6HB stability and thus fusogenicity. A recent study evaluated the structure of spike post-fusion bundle among SARS-CoV-2 variants and showed a slightly displaced 6HB structure of the protein containing the three mutations compared to that of the wildtype protein^28^. The Q954H mutation is not predicted to perturb the 6HB structure as both amino acids can form hydrogen bond with S1175 of HR2 of another S2 (Fig. 6f). Our NMR data above also did not show any notable effect of Q954H on the 6HB stability. Even though it may appear possible that under the acidic pH condition of the endosome, the H954 can be protonated and affect the 6HB structure, our solution NMR data did not show any perturbation of its pK_a_. The N969K mutation would likely perturb the 6HB structure because of the substantial different side chains of asparagine and lysine. In the 6HB structure, the side chain of asparagine (wildtype) can form a pair of hydrogen bonds with the backbone oxygen and nitrogen of G1167, which may form a single hydrogen bond with the protonated side chain of lysine (mutant) (Fig. 6f). Since the L981F and N969K mutations have opposite effects on the fusion activity, the L981F’s effect on the 6HB structure probably counteracts that of N969K, resulting in only a slight displacement with both mutations in the resolved structure^28^. In the currently available structure of the 6HB, the L981F side chain density is not well resolved and thus difficult to say with certainty any impact of the mutation^28^. However, we suggest that the S1161 of HR2 may form a hydrogen bond with the backbone oxygen of L981 but not F981 (Fig. 6f).

## Discussion

Since the end of 2021, the Omicron (BA.1) and its subvariants have emerged and spread worldwide and became the dominant variants^33–35^. Because of their rapid spread, the BA.1 variants were thought to be more infectious and transmissible. However, in vitro studies, while still controversial, have not shown any clear increased infectivity, like the earlier lineages. In fact, BA.1 appears to be less infectious in many studies^13,36^. The increased transmissibility of BA.1 may be a result of increased affinity of the spike protein to ACE2 receptor and/or reduced susceptibility to pre-existing antibodies in the population^37–40^. Indeed, many mutations (>30) are present in the spike protein and distributed widely in the S1 protein, including the N-terminal and receptor binding domains and the S2 protein^11,12,41^. On the other hand, BA.1 appears to exhibit attenuated pathogenicity compared to the earlier lineages^37–40^. Here we showed that BA.1 has significantly lower fusogenicity than all the earlier lineages, including B.1.1.7, B.1.351, B.1.617.2 variants. We reasoned that the reduced fusogenicity likely contributes to the lower pathogenicity of BA.1. Previous studies have supported that the fusogenic property of SARS-CoV-2 is one of the factors contributing to the pathogenesis of SARS-CoV-2^42,43^. A recent report suggests that spike-mediated fusion activates the Caspase-9 pathway, which results in the activation of Caspase-3/7 and gasdermin E-mediated pyroptosis^43^.

In this study, we examined BA.1-associated mutations in the spike protein that may be responsible for its attenuated fusogenic phenotype. We reasoned that mutations in the S1 protein are likely associated with increased ACE2 binding and antibody escape. However, it is possible that mutations in the S1 protein may affect fusogenicity. Indeed, some of the mutations in the S1 protein were reported to be involved not only in increased ACE2 binding and antibody escape, but also in fusogenicity^22,44–46^. Although some of the mutations in the S1 protein may be involved in fusogenicity, we decided to focus on 9 mutations that are located in the subdomain 1/2, around the S1/S2 cleavage site, or in the S2 sequence including HR1, because these mutations were commonly observed in many of the BA.1 subvariants. We reasoned that by studying these mutations, we may gain a mechanistic insight into the fusogenicity and pathogenicity of BA.1 and its subvariants. D614G and P681H were not studied because these two mutations have already been well characterized. D614G mutation enhances infectivity, while fusogenicity is comparable to the level of the parental Wuhan strain^47,48^. The P681H mutation is reported to contribute to increased fusion activity^22^.

Of the nine mutations, we found that five (T547K, H655Y, N856K, Q954H, and N969K) contribute to the attenuated fusogenicity of BA.1. Interestingly, one mutation (L981F) showed an opposite fusogenic effect, which reduces the overall fusogenicity of the combined five mutations. This mutation could have emerged to compensate for the much-reduced fusogenicity of other mutations so the virus would still be infectious. We observed that the five mutations responsible for attenuated fusogenicity showed lower S1/S2 cleavage efficiency that may be explained by structural modeling. A recent paper showed that the H655Y mutation confers a higher fusogenic activity and increased S1/S2 cleavage^9^, which are different from our findings. In that study, the authors tested only one cell line. In our study here, we tested in multiple cell lines with repeated experiments. Thus, we are not sure of the difference.

As described above, the N856K, Q954H, N969K mutations may affect the structure of the S protein in the prefusion state, resulting in reduced S1/S2 cleavage. Based on NMR and CD analyses, the Q954H mutation may also exert another effect, i.e., shifting the equilibrium distribution of the lipid-bound intermediate structural ensemble of HR1^25^, which may adversely impact viral fusion. The overall effect of Q954H, N969K, and L981F on viral fusion may be more complicated. In addition to their effect on S1/S2 cleavage, they may affect the fusion process at the requisite step of forming the 6HB post-fusion structure. Our findings collectively suggest that the effects of these mutations are complicated and intertwined. Some of the mutations may have an epistatic effect in the context of other mutations.

The fusogenic activities of B.1.1.7, B.1.351, and B.1.617.2 variants are proportional to their high infectivities. BA.1 behaves differently compared to the earlier lineages with many mutations emerging in both the S1 and S2 domains. Mutations in the S1 domain likely contribute to the enhanced affinity between spike and ACE2, which would compensate for the attenuated fusogenicity imparted by mutations in the S2 domain. The infectivity of other BA.1 subvariants seem to be lower than that of Omicron BA.1 based on our pseudotyped virus system (Supplementary Fig. 5). Comparison of the sequences of these BA.1 variants (Supplementary Fig. 1) indicates that BA.1 has a unique mutation pattern in the S1 domain compared to the other subvariants, which may explain the higher infectivity of BA.1.

Emerged SARS-CoV-2 variants appeared to be less susceptible to the approved neutralizing monoclonal antibodies targeting the RBD of S1 domain partly because of escape mutations in this region^49^. Recent efforts in developing therapeutic antibodies against SARS-CoV-2 have been focusing on the more conserved fusion peptide/stem helix structure of S2 domain, which mediates viral fusion^50,51^. It is tempting to speculate that Omicron variants with numerous mutations affecting the fusion process may have emerged in response to naturally occurring antibodies against S2 in infected populations and possibly be less sensitive to this alternative class of neutralizing antibodies.

Our study reveals an intriguing and yet complex interplay of various mutations in the spike protein of the BA.1 variants, which have become the dominant viral variants world-wide. These variants likely emerged in the setting of increasing and emerging immunity against the virus, induced either by population-based vaccination or widespread infection, among the world population after two and half years of the pandemic. The evolutionary pressure expectedly selected out variants that are less susceptible to existing immunity, but interestingly also variants, like the Omicrons, that are less virulent. Influenza virus has been known to mutate the furin cleavage site of its hemagglutinin to alter viral tropism and pathogenicity^52^. It is interesting to speculate that less virulent SARS-CoV-2 variants may be more transmissible at the population level because infected people are less symptomatic and aware of their infection, and thus more readily transmit to others, especially in the face of less stringent infection-control precautions over the last year. SARS-CoV-2 may continue to evolve and become an even less virulent virus, like the common cold virus, that will mutate, adapt and spread in the population. It is interesting to speculate that a highly pathogenic virus, once introduced into a susceptible host population, can co-evolve with the host to establish more of a permanent foothold in the microbial universe of the host.

## Methods

### Cells, chemicals, and antibodies

HeLa, Vero E6, and CaCo-2 cells (HeLa, CCL-2; Vero E6, CRL-1586; CaCo-2, HTB-37; ATCC, Manassas, VA, USA) were cultured in EMEM (ATCC, Manassas, VA, USA) and HEK293T cells (CRL-3216, ATCC, Manassas, VA, USA) were maintained in DMEM (Corning, Corning, NY, USA). 293ACE2 (ACE2 expressing cells)^53^ and Huh7.5-A2T2 (ACE2 and TMPRSS2 expressing cells, provided by Charles Rice)^54^ cells were cultured in DMEM (Corning) with 1 µg/mL of puromycin for 293ACE2 and 250 µg/mL of G418 for Huh7.5-A2T2. Vero E6-T2 (TMPRSS2-expressing cells, provided by the SARS-CoV-2 core facility at National Institute of Allergy and Infectious Diseases, National Institutes of Health) cells were maintained in EMEM (ATCC) with 200 µg/mL of Hygromycin B. All media were supplemented with 10% heat-inactivated fetal bovine serum (MilliporeSigma, Burlington, MA, USA) except for CaCo-2 (20% heat-inactivated fetal bovine serum) and the cells were maintained in a 37 °C and 5% CO_2_ incubator.

Chemical compounds were purchased from commercial sources: 25-HC (MilliporeSigma), itraconazole (MilliporeSigma). Antibodies against SARS-CoV-2 S protein were purchased from various commercial sources (anti-S1, Cat#: GTX135356, GeneTex, Irvine, CA, USA and anti-S2, Cat#: PA5-116917, Thermo Fisher Scientific, Waltham, MA, USA, Cat#: GTX632604, GeneTex) and used for various virologic assays. Anti-β-actin (Cat#: ab8227, Abcam, Cambridge, UK), anti-VSV (Cat#: REA005, Imanis Life Sciences, Rochester, MN, USA) antibodies were also obtained commercially.

### Plasmid construction

SARS-CoV-2 S (Genscript, Piscataway NJ, USA) cDNA plasmid was purchased from a commercial source. The C-terminal of the SARS-CoV-2 S sequence (containing an ER retention signal) was truncated by 20 amino acids to increase virus yield^15,55^. In order to introduce a stop codon for truncation, a single nucleotide mutation was made at nucleotide 3759 (C to A) using In-Fusion cloning kit (Takara, Shiga, Japan) according to manufacturer’s instruction. The truncated S sequence was used to replace the VSV-G sequence in the pCMV-VSV-G (Addgene plasmid number: 8454)^56^ plasmid after digestion with BamHI. The mutations in the S sequence of B.1.1.7^4^, B.1.351^57^, B.1.617.2^58^ variants and all the individual S mutants were introduced using Q5 Site-Directed Mutagenesis Kit (New England BioLabs, Ipswich, MA, USA) except for BA.1^11,12,41^ which was synthesized by a commercial source (Genscript). The assembled plasmids were used for VSV pseudotyped virus generation. Full-length (untruncated) S constructs were similarly generated. The mutation sites for B.1.1.7, B.1.351, B.1.617.2, BA.1, and its subvariants (BA.2, BA.2.12.1, BA4, BA.5, and BQ.1)^35,41^ are shown in Supplementary Fig. 1.

For cell-cell fusion assays, additional sequences were added to the above SARS-CoV-2 S constructs to generate both truncated S-SmBit and truncated S-GFP. The BamHI-digested pCMV-VSV-G backbone was also used for both constructs. For truncated S-SmBit construction, the truncated S sequence and P2A-SmBit sequence were amplified by PCR and combined with the digested backbone using the In-Fusion cloning kit according to manufacturer’s instruction. For truncated S-GFP construct generation, the truncated S and P2A sequence from the above construct and GFP were amplified by PCR and combined as above. RFP and LgBit expressing constructs were produced similarly using In-Fusion cloning kit like the above constructions.

### Cell-cell fusion assay

To measure SARS-CoV-2 S protein-mediated cell-cell fusion, HeLa cells (donor) were transfected with S fused with GFP expressing plasmid or truncated S fused with SmBit expressing plasmid^14^. SmBit is from the NanoBit system (Promega, Madison, WI, USA) and pairs with LgBit for emitting luminescence signal. 293ACE2 cells (recipient) were transfected with RFP or LgBit expressing plasmid. FuGENE® 6 Transfection Reagent (Promega) was used for transfection based on manufacturer’s instructions. Colocalization between GFP and RFP allows the visualization of the fusion events and interaction between SmBit and LgBit proteins in fused cells provides a functional enzyme that emits luminescent signals. This SmBit-LgBit system is suitable for the quantification of fusion events^14^. As a negative control, a GFP-SmBit construct was used instead of S-SmBit. Briefly, donor cells and recipient cells were transfected with the appropriate plasmids mentioned above. 24 h post-transfection, the donor and the recipient cells were detached by 5 mM EDTA and Accutase, respectively. For each well of a 96-well plate, 30,000 donor cells and 15,000 recipient cells were seeded. Clear-bottom black plates (Corning) were used for fluorescence measurement and white plates (Greiner Bio-One, Kremsmunster, Austria) were used for luminescence measurement. Then, both cells were cocultured with inhibitor compounds in DMEM containing 10% FBS for 48 h. For GFP-RFP colocalization analysis, 15–20 fields were randomly chosen from 4 replicates to measure the fused cells under a CellSens fluorescence microscope (Olympus, Tokyo, Japan). To quantify colocalization signals, ImageJ (National Institutes of Health, Bethesda, MD, USA) was used. For SmBit-LgBit evaluation, Nanoluciferase substrates (Furimazine) were added and the luminescence signals were measured by a POLARstar Omega plate reader (BMG LABTECH, Ortenberg, Germany) immediately after substrates addition. Background signal from the GFP-SmBit control was subtracted from all experimental samples.

### Viral propagation and infection

The viral stocks were generated, maintained, and handled based on the SOPs formulated by National Institutes of Health in an appropriate designated biosafety-level laboratory. The SARS-CoV-2 variants used in this study were provided by the SARS-CoV-2 core facility of the National Institute of Allergy and Infectious Diseases, National Institutes of Health, and BEI resources (beiresources.org). The references of the variants are as follows: SVG-001/USA-WA1 (Wuhan); SVG-015 UK/CA B.1.1.7; SVG-019 RSA 1.351 501Y; SVG-028 B.1.617.2; SVG −053 BA.1 SARS‐CoV‐ 2/human/U SA/HI‐CDC‐4359259‐001/2021. All the variants above were generated in Vero E6-T2, which expresses TMPRSS2.

For live virus fusion assays, Huh7.5-A2T2 expressing GFP, which was transduced by lentiviral vector were seeded in 96-well plates at a density of 40,000 cells per well. After 24 h, the cells were infected with different SARS-CoV-2 variants and were incubated at 37 °C for 2 h. 2 h post-infection, the cells were washed with phosphate-buffered saline (PBS). 24 h post-infection, the cells were fixed in 4% PFA (ChemCruz, Dallas, TX, USA) for downstream experiments.

### Generation of pseudotyped viruses

A recombinant VSV pseudotyped with its own VSV-G glycoprotein in trans was provided by Adolfo Garcia-Sastre (Mount Sinai School of Medicine, New York, NY, USA)^59^. The native G protein sequence was replaced by the Firefly luciferase, resulting in the replication defective recombinant virus. To produce pseudovirus, the deleted native G glycoprotein was complemented with a VSV-G expressing plasmid.

For generating various pseudotyped viruses, HEK293T cells were seeded at a density of 4 million cells per 10-cm dish and transfected with 10 µg of SARS-CoV-2 variants S expressing plasmids. FuGENE® 6 Transfection Reagent (Promega) was used for transfection based on manufacturer’s instructions. 24 h post-transfection, the cells were infected with the VSV-G pseudotyped viruses and the medium was washed and changed to fresh medium 4 h after post-infection. The SARS-CoV-2 variants S pseudotyped viruses were collected at 24 h post-infection. The virus-containing medium was filtered through a 0.22 µm syringe filter and was stored at −80 °C.

For the pseudotyped virus infectivity assay, target cells were seeded in white 96-well plates at a density of 15,000 cells per well. Before infection, VSV L mRNA levels were measured to calculate genome copy numbers per volume of pseudotyped VSV stock. Then, the cells were inoculated with the same genome copy numbers. 24 h post-seeding, the cells were infected with the same amount of pseudotyped viruses. 24 h postinfection, the cells were lysed in Promega reporter lysis buffer (Promega) for 30 mins followed by freeze-thaw cycles to −80 °C. Amplite™ Luciferase Reporter Gene Assay Kit (AAT Bioquest, Sunnyvale, CA, USA) was used for luciferase activity measurement according to manufacturer’s instructions. The luminescence signals were measured by a POLARstar Omega plate reader (BMG LABTECH).

### Western blotting assay

For protein extraction, RIPA Lysis and Extraction Buffer (Thermo Fisher Scientific) and Protease Inhibitor Cocktail (Roche, Basel, Switzerland) were mixed and used for lysis of cell samples for 10 mins at room temperature. The lysed samples were centrifuged for 10 mins at 12,000 rpm at 4 °C and the supernatants were isolated. The supernatants were used for protein quantification by Pierce™ BCA Protein Assay Kit (Thermo Fisher Scientific). To quantify target protein, automated Western blot was performed on a Simple Western™ Automated Western Blot System (Bio-Techne, Minneapolis, MN, USA) according to the manufacturer’s instructions. All uncropped image data are shown in Supplementary Fig. 9.

### Recombinant protein expression and purification

Gene inserts for the Q954H variant of SARS-CoV-2 Core (HR1 + HR2) and HR1 (Fig. 5a) were synthesized and cloned into the pJ414 vector (ATUM) as described previously^25^. WT and mutants were grown in Escherichia coli BL21 (DE3) at 37 °C and induced for expression at an optical density (600 nm) of 0.8 with a final concentration of 2 mM isopropyl β-D-1-thiogalactopyranoside for 4 hours. HR1 WT and its Q954H variant were purified using Ni-nitriloacetic acid (Ni-NTA) affinity, and reverse phase high-pressure liquid chromatography techniques, which also include an N-terminal His_6_-tag removal by the TEV protease, as described previously^25^. Core WT and its Q954H variant were purified under denaturing conditions by Ni-NTA affinity chromatography and folded on a size-exclusion chromatography column (Superdex-200) in a non-denaturing buffer (20 mM sodium phosphate buffer (pH 6), 150 mM sodium chloride, and 5 mM Imidazole)^25^. Core constructs retained a His_6_-tag at the N-terminus.

### Circular dichroism

Far-UV CD spectra were acquired on a JASCO J-810 spectropolarimeter using a 0.1-cm pathlength cuvette in 20 mM sodium phosphate buffer (pH 6), containing 30 mM sodium chloride. For samples of Core, which did not denature at temperatures below 100 °C, stability was probed after adding 4 M urea. Measurements were performed on 10-μM protein samples. CD melting curves were monitored at 222 nm over the temperature range of 25–95 °C acquired with a ramp rate of 1 °C per minute.

### NMR spectroscopy

HX rates were obtained for two samples containing 0.1 mM [^15^N/^2^H]-HR1^WT^ or [^15^N/^2^H]-HR1^Q954H^ in 20 mM sodium phosphate buffer (at pH 6.5 and pH 7) containing 30 mM sodium chloride and 1 mM imidazole plus 33 mM DMPC and 67 mM DHPC, a lipid mixture that forms disk-shaped mixed micelles commonly referred to as bicelles^60,61^. HX rates were measured using the WEX-III TROSY experiment^62^ using a recycle delay (d1) of 5 s and seven durations of the water inversion interval, ranging from 5 to 1000 ms. Measurements were carried out at 30 °C on an 800 MHz spectrometer. Intrinsic random coil HX rates were obtained from the SPHERE webserver^27^. pH values of the samples were derived from imidazole ^1^H chemical shifts^63^.

The pK_a_ of H954 was determined from a series of ^1^H-^15^N TROSY-HSQC spectra for a sample containing 0.15 mM [^15^N/^2^H]-Core^Q954H^ in 20 mM sodium phosphate buffer and 30 mM NaCl, by stepwise changes of the sample pH over the range of 5.0 to 7.6. The pK_a_ was extracted from ^1^H and ^15^N chemical shifts by using the equation^63^:

1 $${\delta }_{{obs}}={\delta }_{{HA}}+\frac{({\delta }_{{HA}}-{\delta }_{A})}{1+{10}^{\left({{pK}}_{a}-{pH}\right)}}$$

where δ_obs_ is the observed NMR chemical shift of the peak of interest, δ_HA_ and δ_A_ are the chemical shifts of the protonated and deprotonated form.

### Immunofluorescence and confocal microscopy

HeLa cells were transfected with SARS-CoV-2 variants S plasmids and were fixed with 4% paraformaldehyde (ChemCruz) 48 h post-transfection. After fixation, the cells were then permeabilized with 0.5% Triton X-100 in PBS and were blocked by 3% bovine serum albumin (BSA, MilliporeSigma) in PBS. Anti-S2 (Cat#: GTX632604, GeneTex) were diluted in 0.1% Triton X-100 and 1% BSA containing PBS. Alexa Fluor 555 donkey anti-mouse IgG (Thermo Fisher Scientific) was added to the cells as a secondary antibody. DAPI (Thermo Fisher Scientific) was used for nuclei staining. Images were taken by a Zeiss LSM 700 confocal microscope (Carl Zeiss AG, Oberkochen, Germany).

### ATP bioluminescence assay

The PhosphoWorks™ Luminometric ATP Assay Kit (AAT Bioquest) was used to measure the viability of target cells that were transfected with various SARS-CoV-2 variants S plasmids. HeLa cells were transfected with SARS-CoV-2 variants S plasmids and were transferred to white 96-well plates at a density of 10,000 cells per well 24 h post-transfection. After additional 24 h, luciferase activities were assessed on a POLARstar Omega plate reader (BMG LABTECH).

### In silico structural modeling

The structures of SARS-CoV-2 spike protein of Wuhan and BA.1 were resolved by cryo-EM and retrieved from the Protein Data Bank with the PDB ID 7DZW (Wuhan), 7WK2 (BA.1, S-close), and 7WVN (BA.1, S-open). Prior to modeling analysis, the Wuhan and variant trimer protein structures were refined and superimposed using the MOE program. In addition, the 6HB structure of HR1 and HR2 in the postfusion state has also been characterized^28^. Both the Wuhan (7RZV) and BA.1 (7TIK) were obtained from the PDB and used for structural studies. To define various intermolecular interactions such as hydrogen bond, salt bridge, etc., Discovery Studio Visualizer software was used.

### Statistics and reproducibility

Data were analyzed with GraphPad Prism 9 software (GraphPad Software, San Diego, CA, USA) and were shown as mean (SD) of more than three experimental replicates. Two-sided *P* values, adjusted for one-way ANOVA multiple comparison whenever appropriate, were used in analyses and *P* values < 0.05 were considered to be statistically significant. *P < 0.05, **P < 0.01, ***P < 0.001, ****P < 0.0001. The sample size used to derive each statistical results was shown in the appropriate figure legends.

### Reporting summary

Further information on research design is available in the Nature Portfolio Reporting Summary linked to this article.

## Supplementary information

Supplementary Information

Description of Additional Supplementary Data

Supplementary Data

Reporting Summary

## Data Availability

The numerical source data for graphs can be found in Supplementary Data. The data that support this study are available from the corresponding author upon request.
