# Supplementary material for: SARS-CoV-2 omicron variants harbor spike protein mutations responsible for their attenuated fusogenic phenotype
Source: Commun Biol. 2023 May 24;6:556. doi: 10.1038/s42003-023-04923-x (PMC10206564; doi:10.1038/s42003-023-04923-x)

Supplementary Information

SARS-CoV-2 Omicron Variants Harbor Spike Protein Mutations Responsible for Attenuated Fusogenic Phenotype

Seung Bum Park[1, *], Mohsin Khan[1], Sai Chaitanya Chiliveri[2], Xin Hu[3], Parker Irvin[1], Madeleine Leek[1], Ailis Grieshaber[1], Zongyi Hu[1], Eun Sun Jang[1, 4], Ad Bax[2], and T. Jake Liang[1, *]

[1]Liver Diseases Branch, National Institute of Diabetes and Digestive and Kidney Diseases (NIDDK), National Institutes of Health, Bethesda, MD, 20892, USA

[2]Laboratory of Chemical Physics, National Institute of Diabetes and Digestive and Kidney Diseases (NIDDK), National Institutes of Health, Bethesda, MD, 20892, USA.

[3]National Center for Advancing Translational Sciences (NCATS), National Institutes of Health, Rockville, MD, 20850, USA

[4]Department of Internal Medicine, Seoul National University Bundang Hospital, Seoul National University College of Medicine, Seongnam, 13620, Republic of Korea

[*]To whom correspondence should be addressed. Tel: 1 (301) 496-1721; Email: seungbum.park@nih.gov or jliang@nih.gov

Present Address: [Seung Bum Park or T. Jake Liang], Liver Diseases Branch, National Institute of Diabetes and Digestive and Kidney Diseases (NIDDK), National Institutes of Health, Building 10, Room 9B16, 10 Center Drive, Bethesda, MD 20892, USA

Supplementary figures

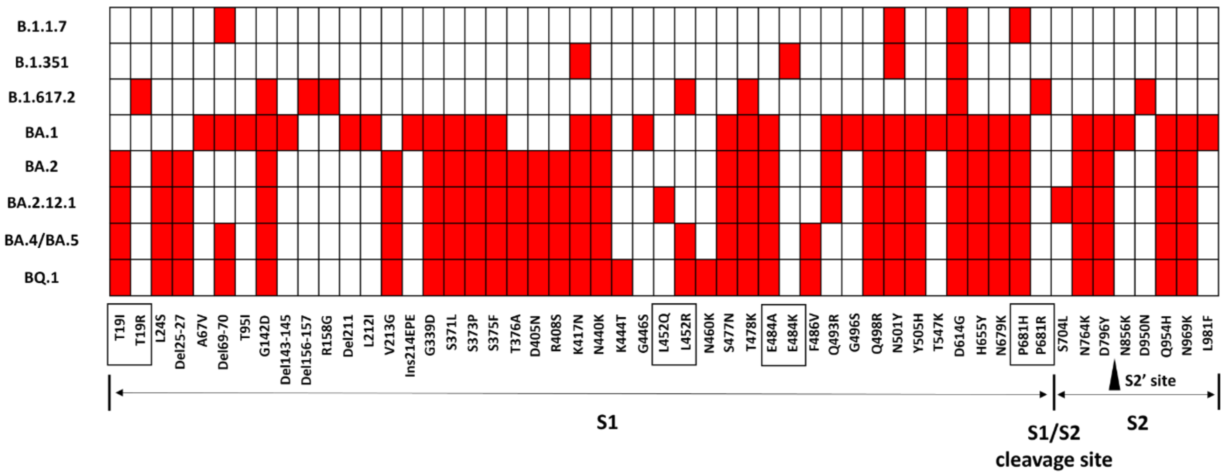

Supplementary Fig. 1. Mutations present in SARS-CoV-2 variants. Comparison of S sequences of SARS-CoV-2 variants versus Wuhan are shown and mutations are highlighted in red. Boxed mutation sites show two different mutations at the same site.

a)

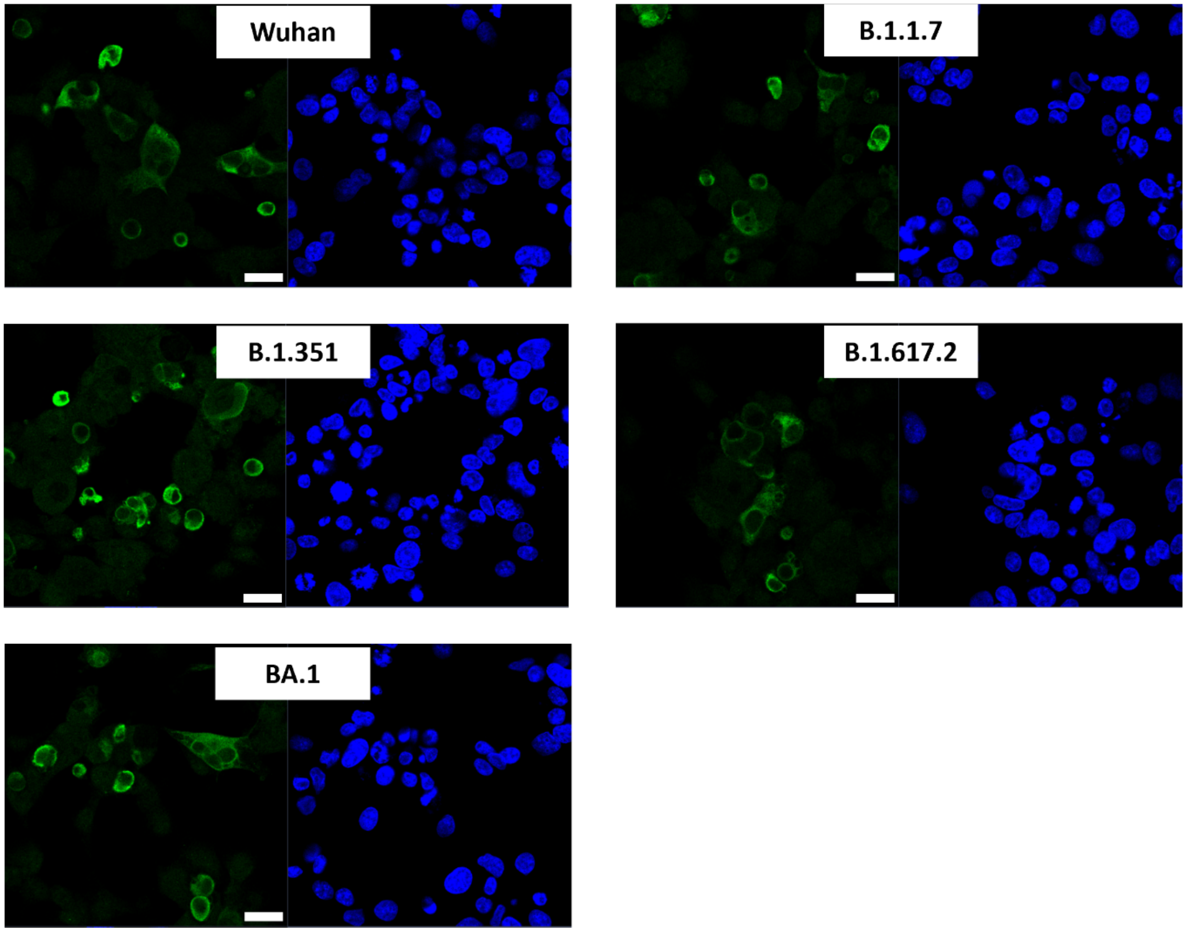
Wuhan
B.1.1.7
B.1.351
B.1.617.2
BA.1

b)

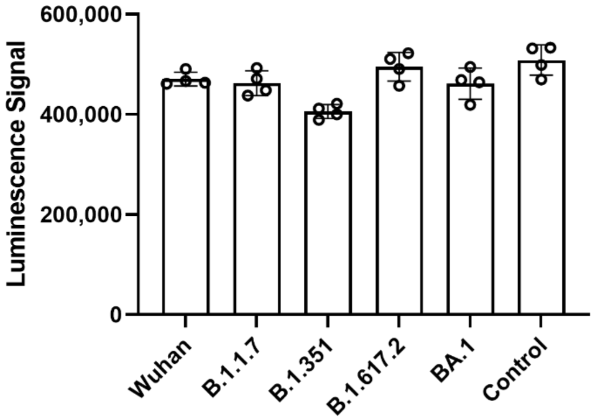
Luminescence Signal
600,000
400,000
200,000
0
Wuhan
B.1.1.7
B.1.351
B.1.617.2
BA.1
Control

Supplementary Fig. 2. Cellular distribution and cytotoxicity of SARS-CoV-2 variant spike proteins. HeLa cells were transfected with SARS-CoV-2 variant S expressing vectors. **a)** 48 h post-transfection, the cells were fixed with 4% PFA followed by permeabilization with 0.5% Triton X-100 in PBS. Then, the cells were blocked by 3% BSA in PBS. Anti-S2 antibody was used to evaluate the distribution of S. For nuclei staining, DAPI was used. Images were taken by Zeiss LSM 700 confocal microscope. Scale bar 50 μm. **b)** Cytotoxicity assay. 24 h post-transfection, cells were transferred to white 96-well plates. After additional 24 h, luminescence signals were assessed on a POLARstar Omega plate reader (BMG LABTECH) using the PhosphoWorks™ Luminometric ATP Assay Kit. All data points are presented as mean values (SD, n=4 biological independent replicates).

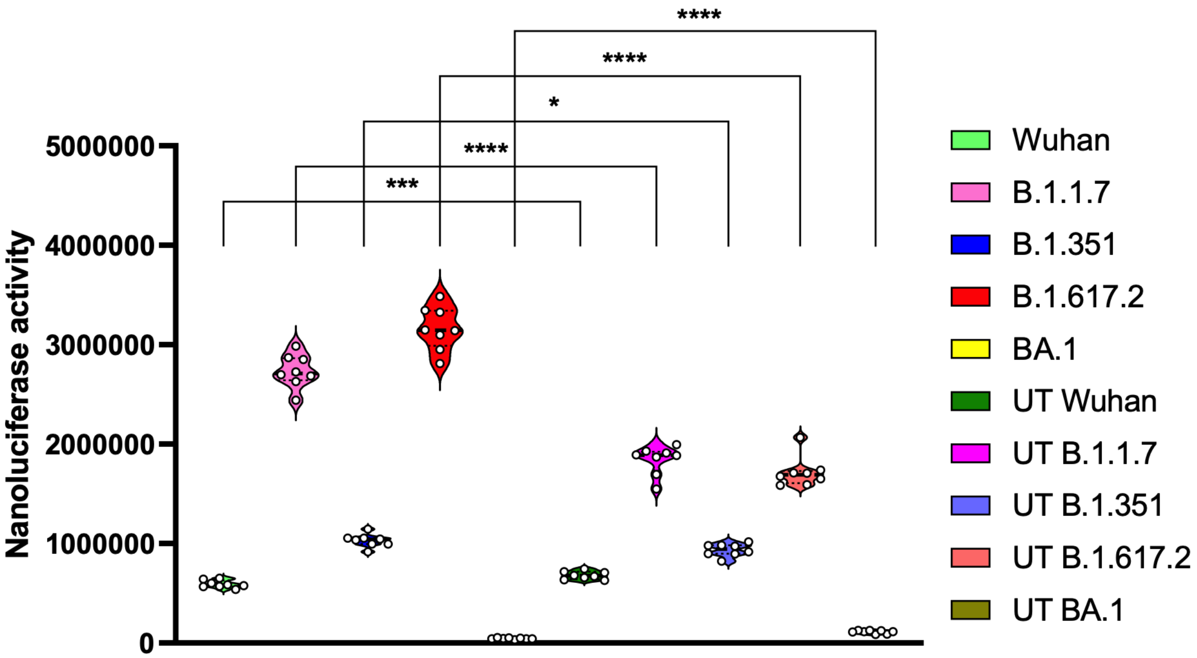

Supplementary Fig. 3. Fusogenic activities of SARS-CoV-2 S variants. Cell-cell fusion assay was performed with SARS-CoV-2 variants using S constructs that are either truncated or untruncated/full-length (UT) (**See Methods**). The various S-SmBit constructs transfected donor (HeLa) cells and the LgBit construct transfected recipient (293ACE2) cells were mixed 24 h post-transfection and were incubated for 48 h. After incubation, luminescence signals were measured by a POLARstar Omega plate reader. All data points are presented as mean values (SD, n=8 biological independent replicates).

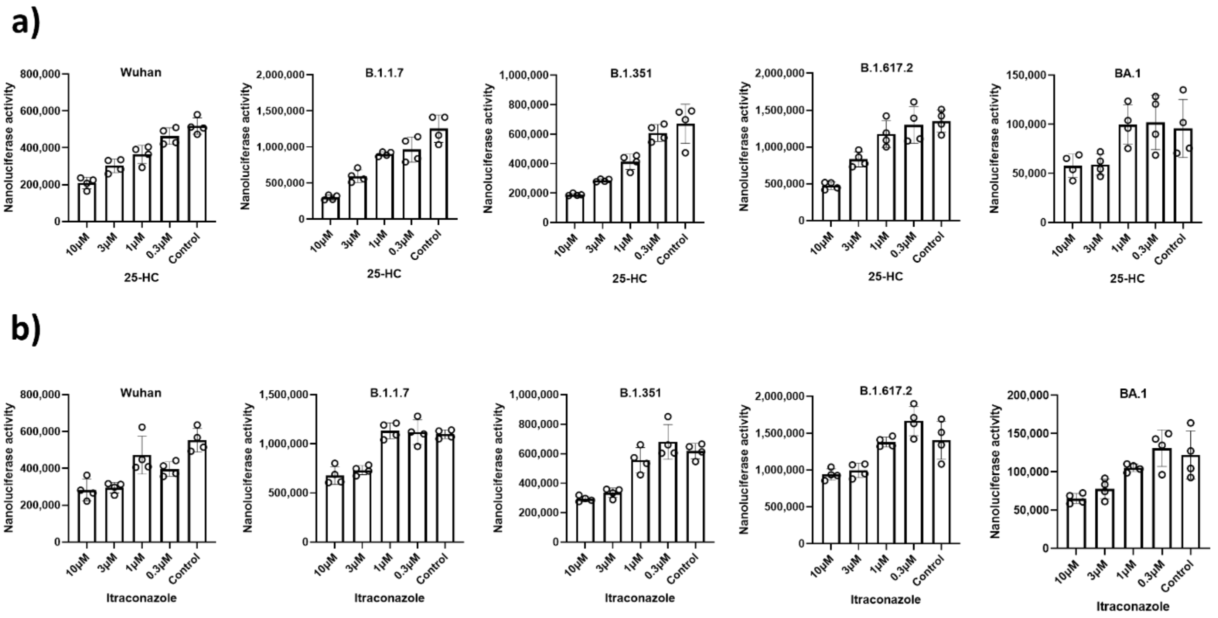

Supplementary Fig. 4. Dose-dependent inhibition of SARS-CoV-2 variants by fusion inhibitors. Cell-cell fusion assays were performed with various SARS-CoV-2 variant S. The truncated S-SmBit transfected donor (HeLa) cells and the LgBit transfected recipient (293ACE2) cells were mixed 24 h post-transfection and treated with four different concentrations (10 μM, 3 μN, 1 μM, 0.3 μM) of 25-HC (**a)** or Itraconazole (**b)**. 48 h post-mixture, luminescence signals were measured by a POLARstar Omega plate reader. All data points are presented as mean values (SD, n=4 biological independent replicates).

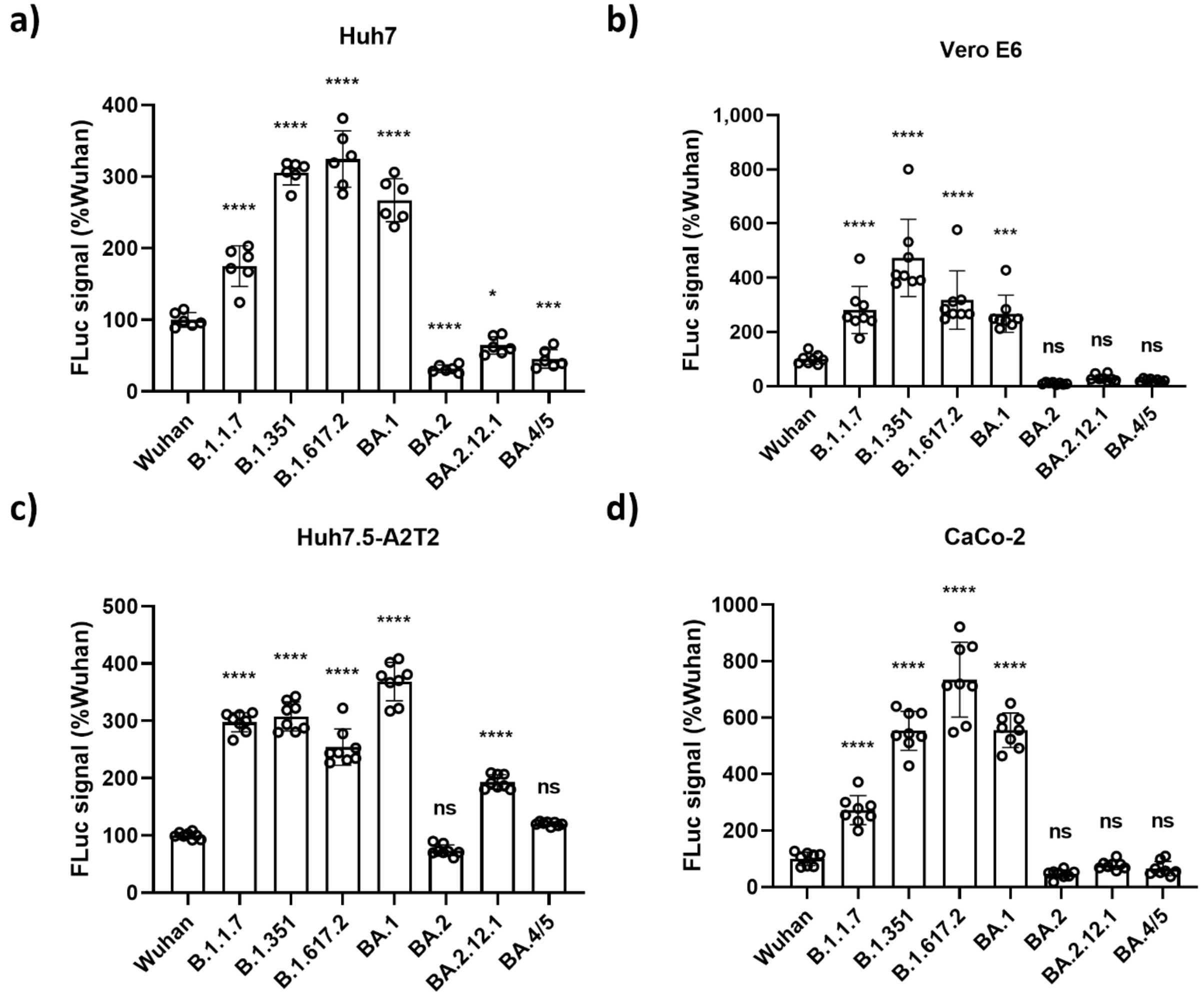

Supplementary Fig. 5. SARS-CoV-2 variant S coated pseudoparticle assay. SARS-CoV-2 variant S coated pseudoparticles were used to infect Huh7 (**a**), Vero E6 (**b**), Huh7.5-A2T2 (**c**), and CaCo-2 (**d**). For the pseudotyped VSV stock, VSV L mRNA levels were quantified to calculate genome copy numbers per volume. Cells were then infected with the same genome copy numbers (4.6×10^{5}). The luminescence signals were measured using a POLARstar Omega plate reader. All data points are presented as mean values (SD, n=6-8 biological independent replicates).

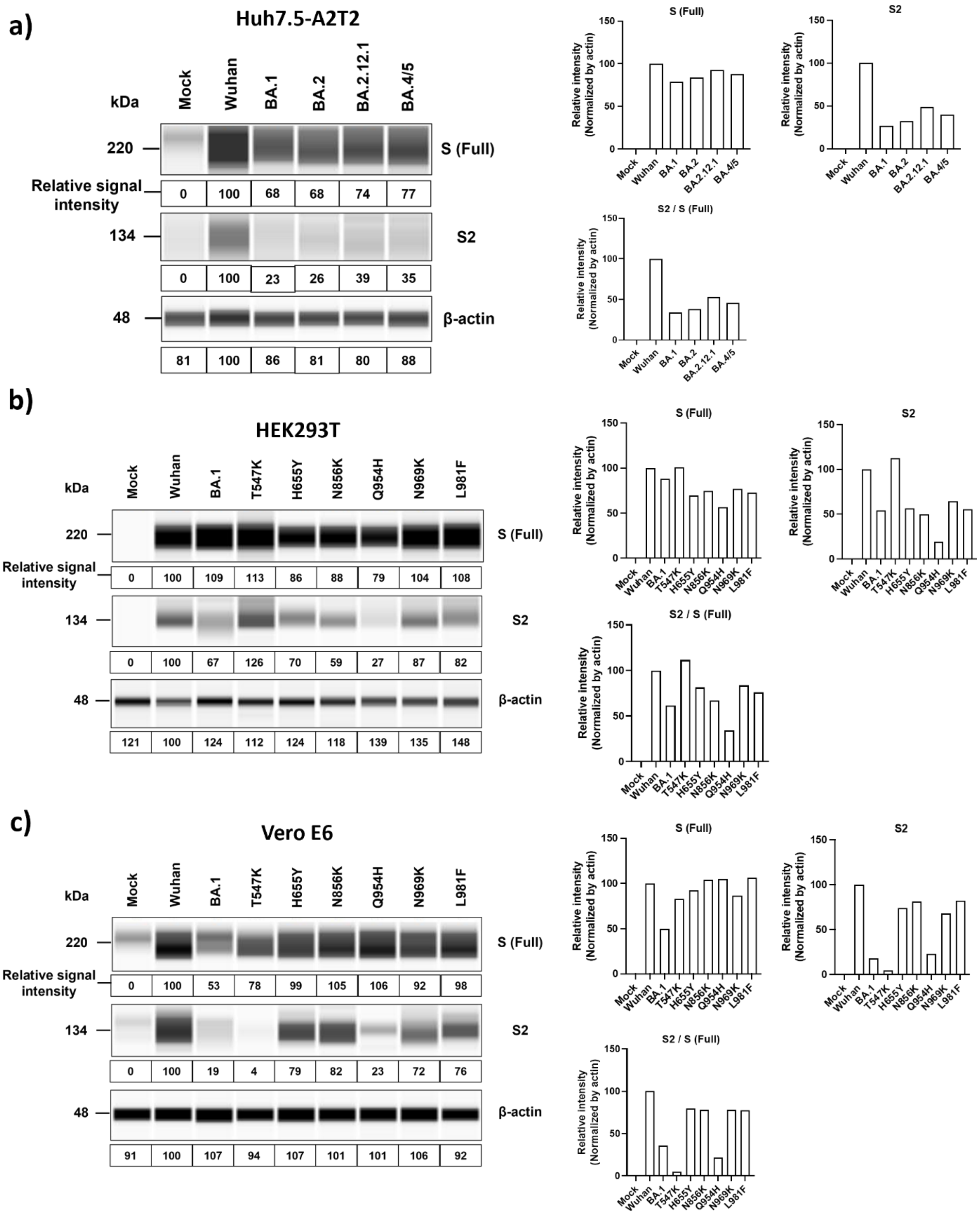
a)
Huh7.5-A2T2
kDa
Mock
Wuhan
BA.1
BA.2
BA.2.12.1
BA.4/5
220
S (Full)
Relative signal intensity
0 100 68 68 74 77
134
S2
0 100 23 26 39 35
48
β-actin
81 100 86 81 80 88
S (Full)
S2
S2 / S (Full)
Relative intensity (Normalized by actin)
b)
HEK293T
Mock
Wuhan
BA.1
T547K
H655Y
N856K
Q954H
N969K
L981F
0 100 109 113 86 88 79 104 108
0 100 67 126 70 59 27 87 82
121 100 124 112 124 118 139 135 148
c)
Vero E6
0 100 53 78 99 105 106 92 98
0 100 19 4 79 82 23 72 76
91 100 107 94 107 101 101 106 92

Supplementary Fig. 6. Effects of BA.1 variant mutations on S1/S2 cleavage in various cell lines. a) Huh7.5-A2T2 cells were transfected with SARS-CoV-2 variants S expressing vectors. BA.1-associated mutants S expressing vectors were transfected into HEK293T **(b)** and Vero E6 **(c)**. 24 h post-transfection, cells were collected with RIPA Lysis and Extraction Buffer and Protease Inhibitor Cocktail. The lysed samples were processed further to measure the S/S2 level with automated Western blot system (Simple Western™ Automated Western Blot System). Then, ImageJ was used to quantify the relative signal intensity. The β-actin-normalized relative signal intensities were plotted separately as S (Full), S2, and S2/S (Full).

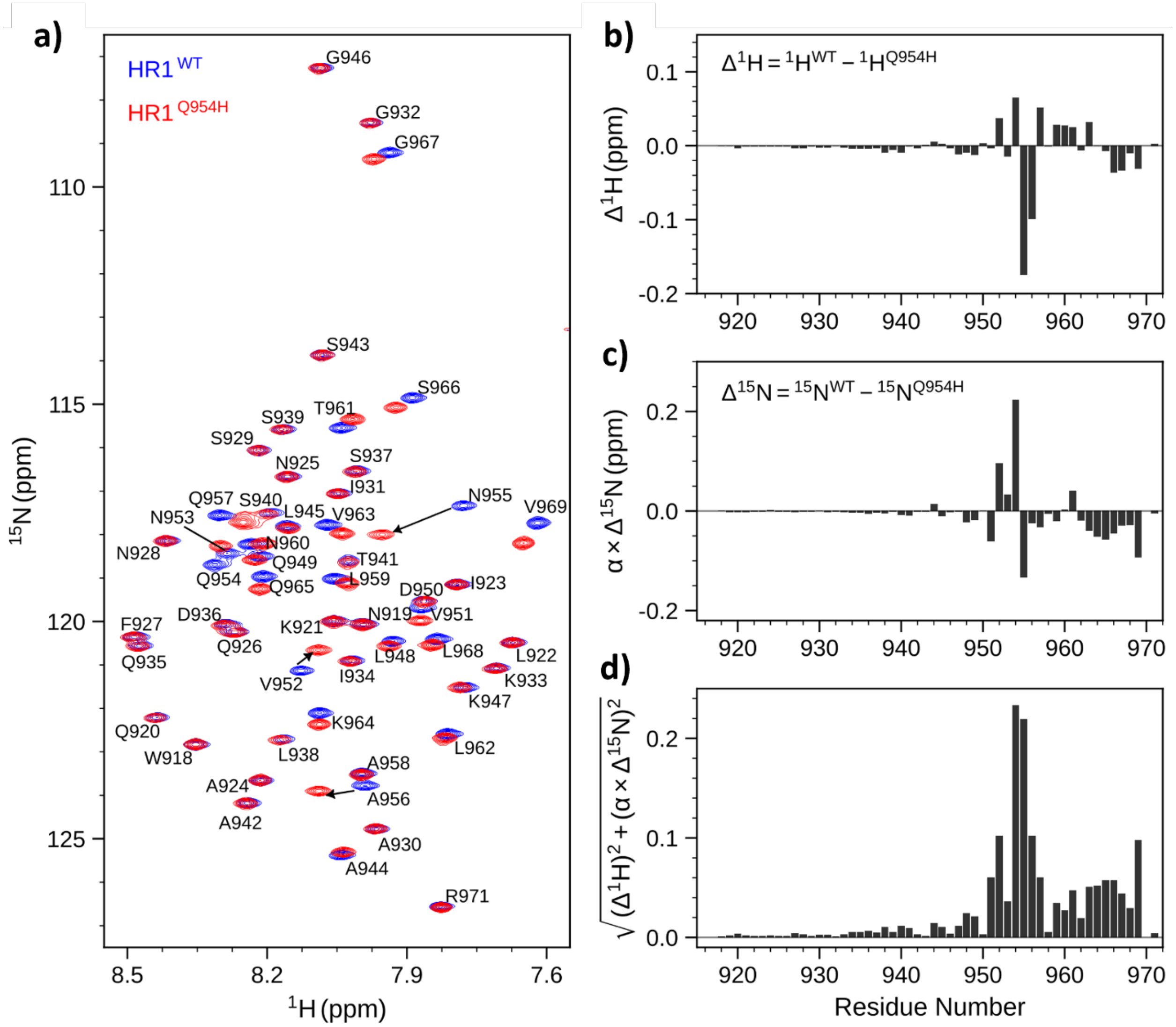

Supplementary Fig. 7. Comparison of the NMR spectra of HR1 WT and Q954H mutant in lipid-bound state. a) Overlay of ^{1}H-^{15}N TROSY-HSQC spectra of the HR1 WT (blue) and Q954H variant (red). Assignments are marked for the wild type. Change in **(b)** ^{1}H, **(c)** ^{15}N (scaling factor, α=0.2), and **(d)** normalized chemical shift differences between the WT and Q954H. Data were obtained on 100 µM HR1 WT and Q954H in the presence of 20 mM sodium phosphate buffer (pH 6) containing 30 mM sodium chloride and bicelles (33 mM dimyristoyl phosphatidylcholine and 67 mM dihexanoyl phosphatidylcholine) at 35 ºC on an 800 MHz NMR spectrometer.

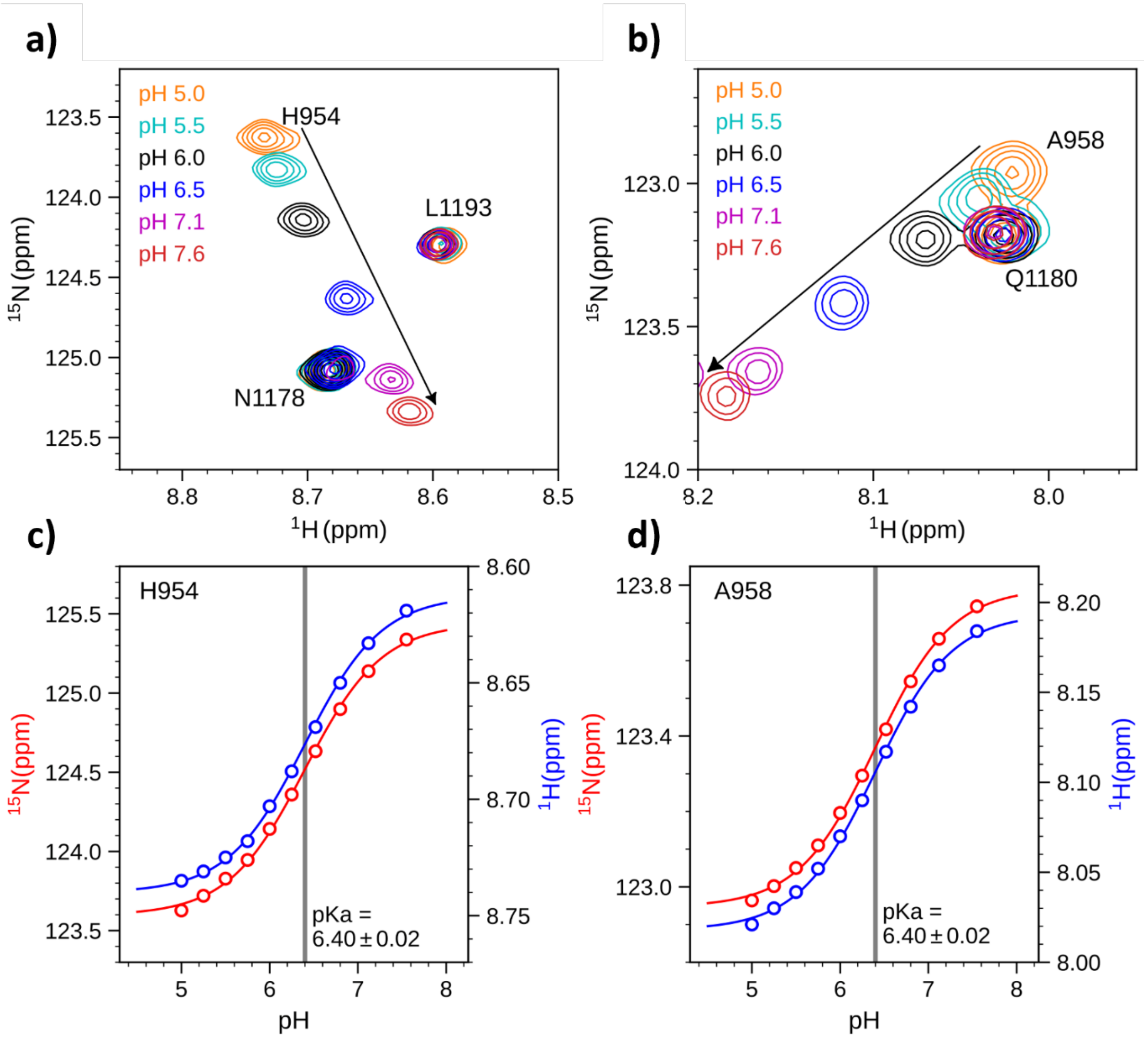

Supplementary Fig. 8. Determining pK$_a$ of H954. Overlay of small regions of ^{1}H-^{15}N TROSY-HSQC spectra depicting chemical shift changes for residues **(a)** H954 and (**b)** A958, by varying the pH over the range of 5.0 to 7.6 (5.0-orange, 5.5-cyan, 6.0-black, 6.5-blue, 7.1-magenta, and 7.6-red). ^{15}N (red circles) and ^{1}H (blue circles) chemical shifts for (**c**) H954 and (**d**) A958 as a function of pH. Global fitting (solid lines) for these four datasets to Eq. 1 resulted in a pK$_a$ of 6.40.

Supplementary Fig. 9. Uncropped images for Western blot assay.

Fig. 4a S2 (Low intensity)

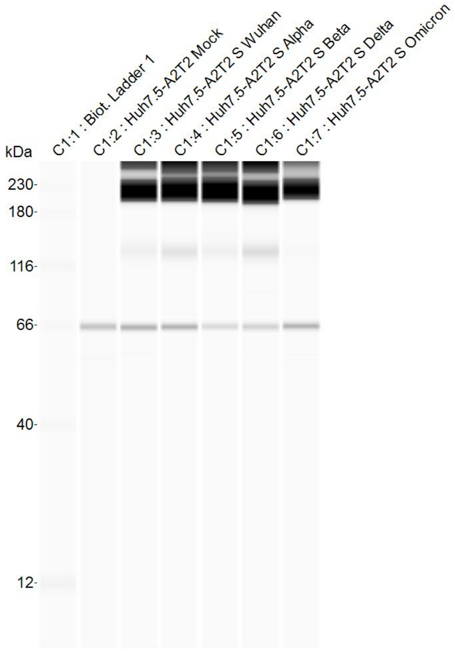

Fig. 4a S2 (High intensity)

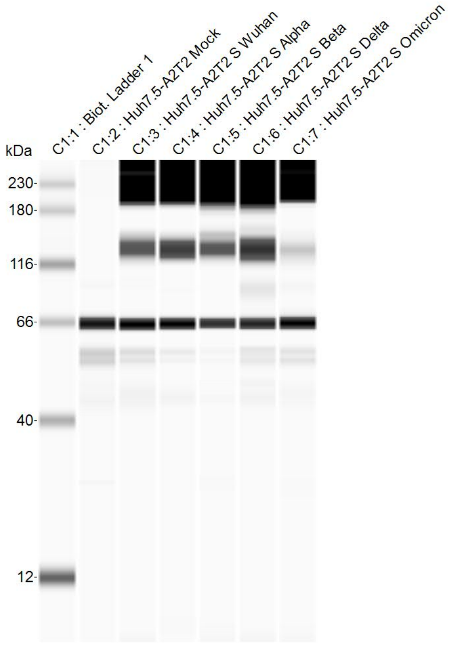

Fig. 4a Actin

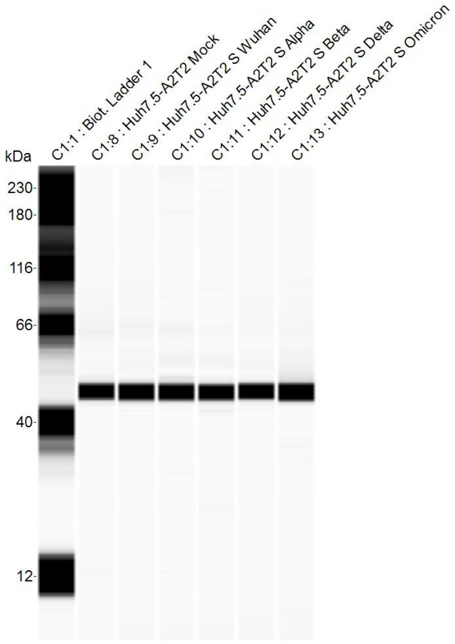

Fig. 4b S2 (Low intensity)

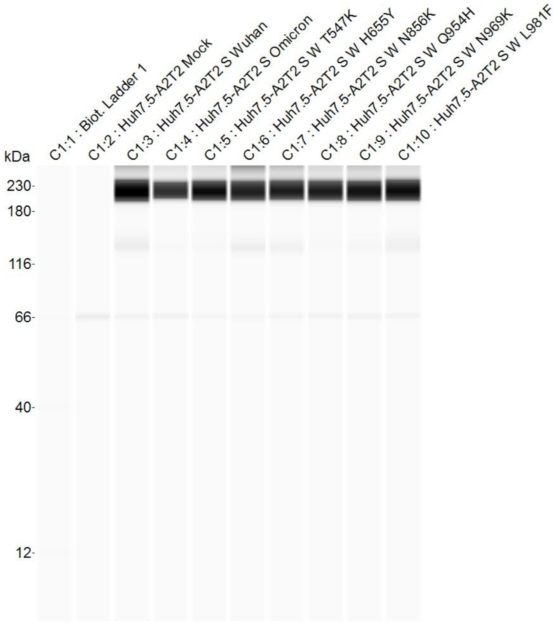

Fig. 4b S2 (High intensity)

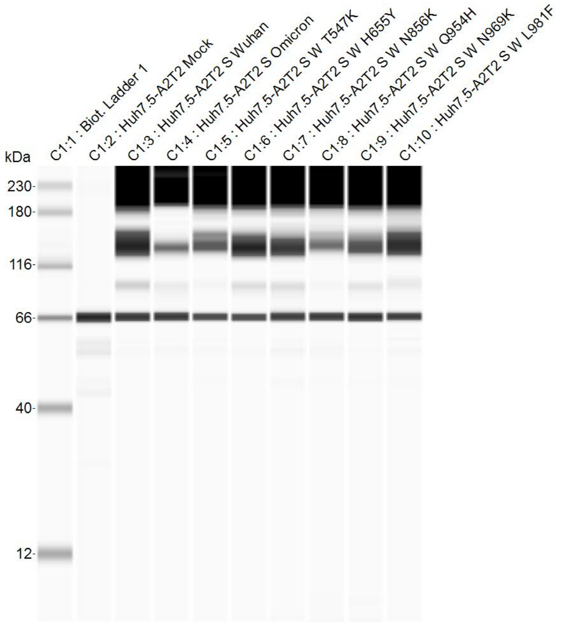

Fig. 4b Actin

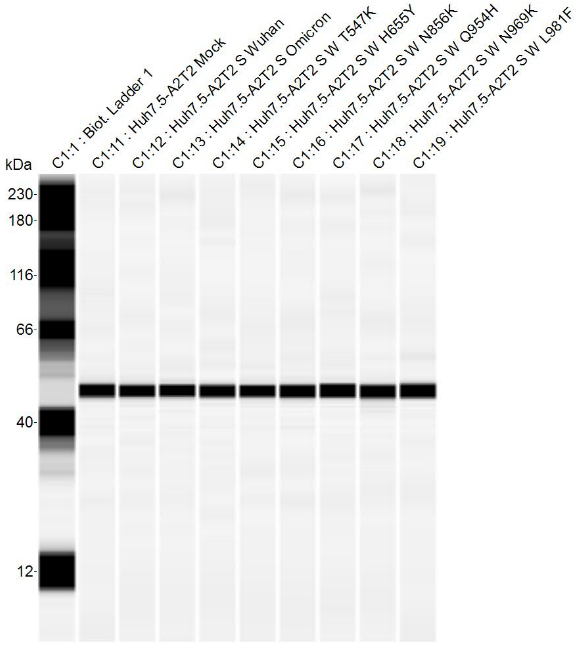

Supplementary Fig. 6a S2 (Low intensity)

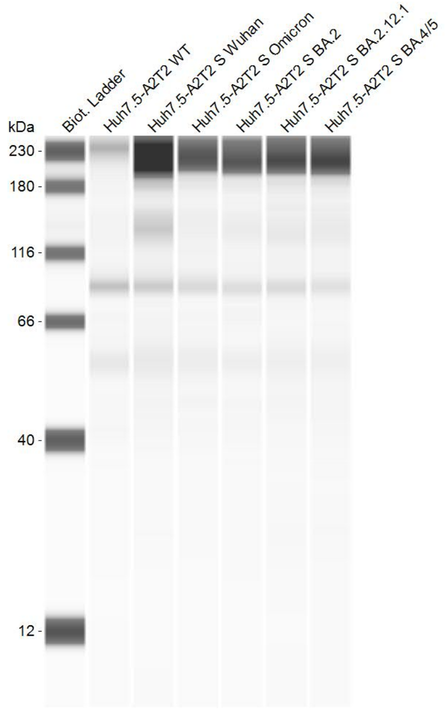

Supplementary Fig. 6a S2 (High intensity)

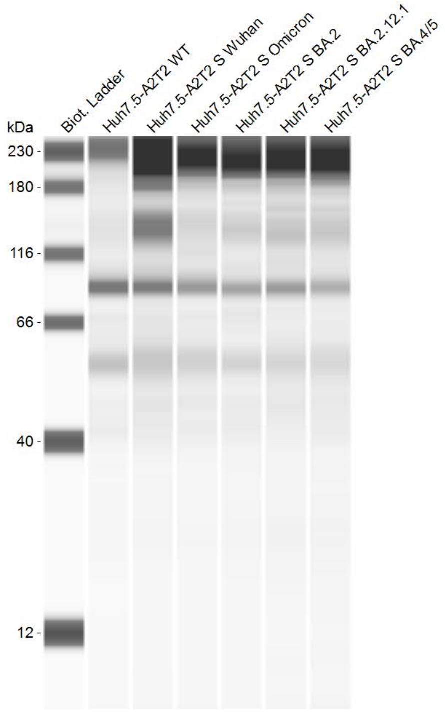

Supplementary Fig. 6a Actin

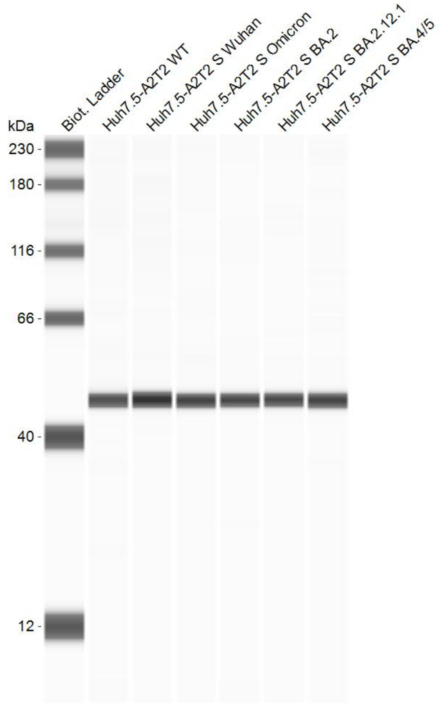

Supplementary Fig. 6b S2 (Low intensity)

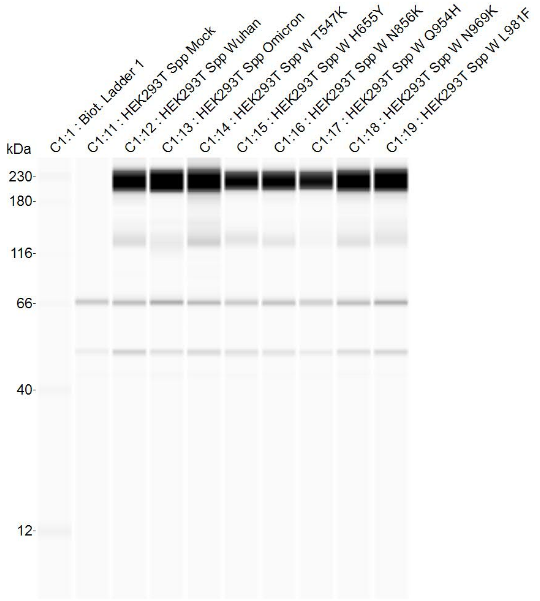

Supplementary Fig. 6b S2 (High intensity)

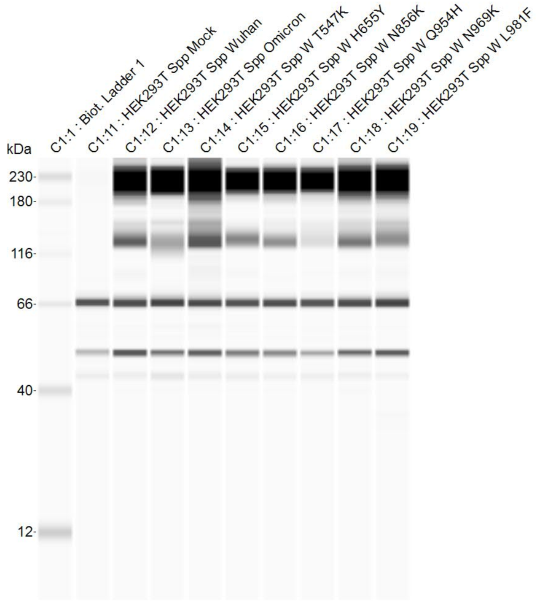

Supplementary Fig. 6b Actin

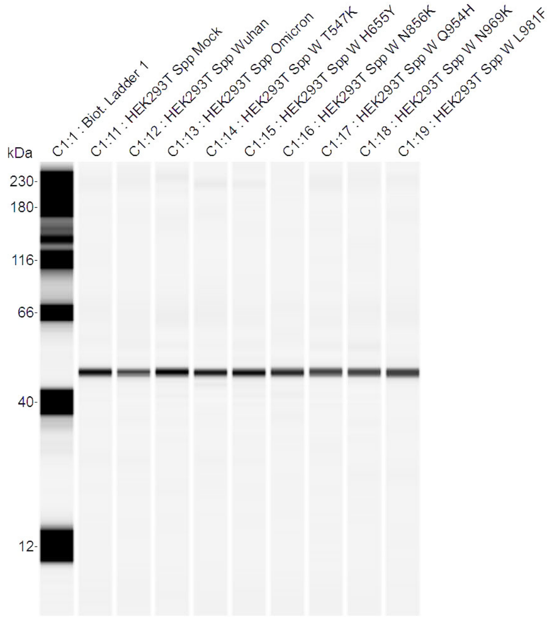

Supplementary Fig. 6c S2 (Low intensity)

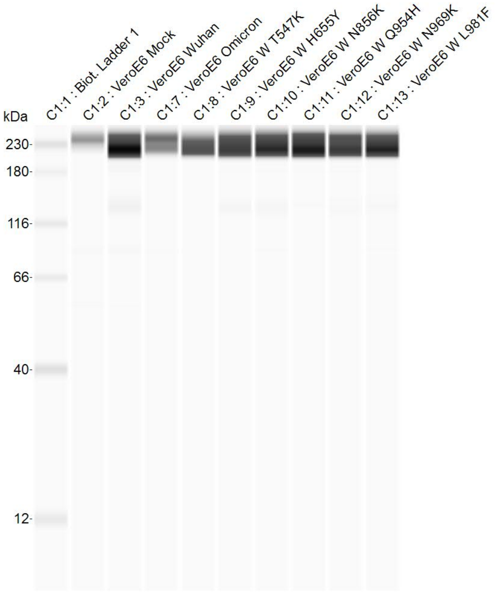

Supplementary Fig. 6c S2 (High intensity)

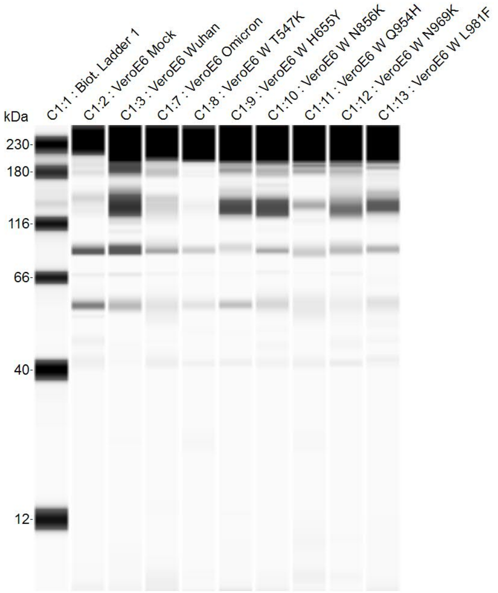

Supplementary Fig. 6c Actin

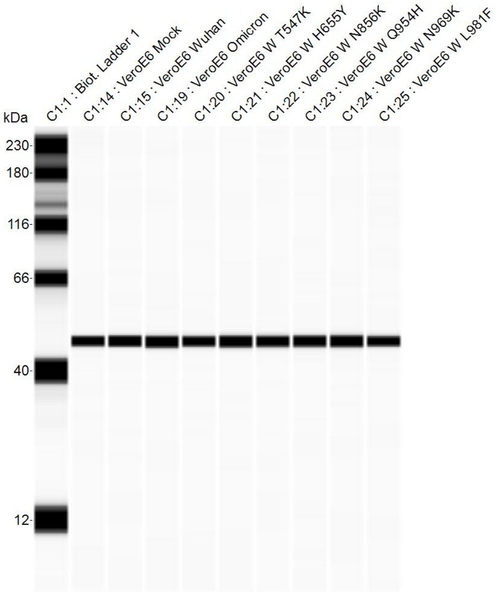

Supplement: Supplementary file 1 — Supplementary Information [file 42003_2023_4923_MOESM1_ESM.pdf]
