# Supplementary material for: SARS-CoV-2 omicron variants harbor spike protein mutations responsible for their attenuated fusogenic phenotype
Source: Commun Biol. 2023 May 24;6:556. doi: 10.1038/s42003-023-04923-x (PMC10206564; doi:10.1038/s42003-023-04923-x)
Supplement: Supplementary file 2 — Description of Additional Supplementary Data [file 42003_2023_4923_MOESM2_ESM.docx]

**Description of Additional Supplementary Files**

**File name:** Supplementary Data

**Description:** It’s the numerical source data for graphs presented in the main figures.
