# Supplementary material for: SARS-CoV-2 omicron variants harbor spike protein mutations responsible for their attenuated fusogenic phenotype
Source: Commun Biol. 2023 May 24;6:556. doi: 10.1038/s42003-023-04923-x (PMC10206564; doi:10.1038/s42003-023-04923-x)
Supplement: Supplementary file 4 — Reporting Summary [file 42003_2023_4923_MOESM4_ESM.pdf]

# nature portfolio

Corresponding author(s): Seung Bum Park and T. Jake Liang

Last updated by author(s): May 3, 2023

# Reporting Summary

Nature Portfolio wishes to improve the reproducibility of the work that we publish. This form provides structure for consistency and transparency in reporting. For further information on Nature Portfolio policies, see our Editorial Policies and the Editorial Policy Checklist.

## Statistics

For all statistical analyses, confirm that the following items are present in the figure legend, table legend, main text, or Methods section.

| n/a | Confirmed | |
|---|---|---|
| ☐ | ☒ | The exact sample size (*n*) for each experimental group/condition, given as a discrete number and unit of measurement |
| ☐ | ☒ | A statement on whether measurements were taken from distinct samples or whether the same sample was measured repeatedly |
| ☐ | ☒ | The statistical test(s) used AND whether they are one- or two-sided<br>*Only common tests should be described solely by name; describe more complex techniques in the Methods section.* |
| ☒ | ☐ | A description of all covariates tested |
| ☒ | ☐ | A description of any assumptions or corrections, such as tests of normality and adjustment for multiple comparisons |
| ☐ | ☒ | A full description of the statistical parameters including central tendency (e.g. means) or other basic estimates (e.g. regression coefficient) AND variation (e.g. standard deviation) or associated estimates of uncertainty (e.g. confidence intervals) |
| ☐ | ☒ | For null hypothesis testing, the test statistic (e.g. *F*, *t*, *r*) with confidence intervals, effect sizes, degrees of freedom and *P* value noted<br>*Give P values as exact values whenever suitable.* |
| ☒ | ☐ | For Bayesian analysis, information on the choice of priors and Markov chain Monte Carlo settings |
| ☒ | ☐ | For hierarchical and complex designs, identification of the appropriate level for tests and full reporting of outcomes |
| ☒ | ☐ | Estimates of effect sizes (e.g. Cohen's *d*, Pearson's *r*), indicating how they were calculated |

*Our web collection on statistics for biologists contains articles on many of the points above.*

## Software and code

Policy information about availability of computer code

| | |
|---|---|
| Data collection | N/A |
| Data analysis | N/A |

For manuscripts utilizing custom algorithms or software that are central to the research but not yet described in published literature, software must be made available to editors and reviewers. We strongly encourage code deposition in a community repository (e.g. GitHub). See the Nature Portfolio guidelines for submitting code & software for further information.

## Data

Policy information about availability of data

All manuscripts must include a data availability statement. This statement should provide the following information, where applicable:

- Accession codes, unique identifiers, or web links for publicly available datasets
- A description of any restrictions on data availability
- For clinical datasets or third party data, please ensure that the statement adheres to our policy

The datasets generated during and/or analysed during the current study are available from the corresponding author on reasonable request.

## Human research participants

Policy information about studies involving human research participants and Sex and Gender in Research.

| | |
|---|---|
| Reporting on sex and gender | N/A |
| Population characteristics | N/A |
| Recruitment | N/A |
| Ethics oversight | N/A |

Note that full information on the approval of the study protocol must also be provided in the manuscript.

# Field-specific reporting

Please select the one below that is the best fit for your research. If you are not sure, read the appropriate sections before making your selection.

- [x] Life sciences
- [ ] Behavioural & social sciences
- [ ] Ecological, evolutionary & environmental sciences

For a reference copy of the document with all sections, see nature.com/documents/nr-reporting-summary-flat.pdf

# Life sciences study design

All studies must disclose on these points even when the disclosure is negative.

| | |
|---|---|
| Sample size | The sample sizes (n>3) for cell culture experiments were chosen for applying statistical tests. |
| Data exclusions | No data were excluded in this study. |
| Replication | The data are representative of at least 2 experiments with multiple samples. |
| Randomization | No method of randomization was used in this study. |
| Blinding | The number of investigators were limited. Therefore, blinding was not carried out. |

# Reporting for specific materials, systems and methods

We require information from authors about some types of materials, experimental systems and methods used in many studies. Here, indicate whether each material, system or method listed is relevant to your study. If you are not sure if a list item applies to your research, read the appropriate section before selecting a response.

### Materials & experimental systems

| n/a | Involved in the study | |
|---|---|---|
| [ ] | [x] | Antibodies |
| [ ] | [x] | Eukaryotic cell lines |
| [x] | [ ] | Palaeontology and archaeology |
| [x] | [ ] | Animals and other organisms |
| [x] | [ ] | Clinical data |
| [x] | [ ] | Dual use research of concern |

### Methods

| n/a | Involved in the study | |
|---|---|---|
| [x] | [ ] | ChIP-seq |
| [x] | [ ] | Flow cytometry |
| [x] | [ ] | MRI-based neuroimaging |

## Antibodies

| | |
|---|---|
| Antibodies used | For automated Western blot system (Wes assay),<br>rabbit anti-SARS-CoV-2 Spike S1 polyclonal antibody (GeneTex, Cat#: GTX135356, 1:100)<br>rabbit anti-SARS-CoV-2 Spike S2 polyclonal antibody (Thermo Fisher Scientific, Cat#: PA5-116917, 1:100)<br>mouse anti-SARS-CoV-2 Spike S2 monoclonal antibody (clone 1A9, GeneTex, Cat#: GTX632604, 1:100)<br>rabbit anti-β-actin polyclonal antibody (Abcam, Cat#: ab8227, 1:100)<br>rabbit anti-VSV polyclonal antibody (Imanis Life Sciences, Cat#: REA005, 1:100) |
| Validation | Validation of all primary antibodies for the species and application was conducted by manufacturers prior to sale, and validation |

statements are available on the manufacturers' website.

## Eukaryotic cell lines

Policy information about cell lines and Sex and Gender in Research

| | |
|---|---|
| Cell line source(s) | HeLa cells (a human epithelial cell line; ATCC CCL-2)<br>Vero E6 cells (an African green monkey kidney cell line; ATCC CRL-1586)<br>HEK293T cells (a human kidney cell line; ATCC CRL-3216)<br>293ACE2 (a human kidney cell line stably expressing ACE2; Zhang et al., Cell Discovery, 2020)<br>Huh7.5-A2T2 (a human hepatocyte cell line stably expressing ACE2 and TMPRSS2; Ricardo-Lax et al., Science, 2021)<br>VeroE6-T2 (an African green monkey kidney cell line stably expressing TMPRSS2; provided by the SARS-CoV-2 core facility at National Institute of Allergy and Infectious Diseases, National Institutes of Health)<br>CaCo-2 cells (a human colon cell line: ATCC HTB-37) |
| Authentication | None of the cells used were authenticated. |
| Mycoplasma contamination | None of the cells used were tested for mycoplasma contamination. |
| Commonly misidentified lines<br>(See ICLAC register) | No commonly misidentified cell lines were used. |
